# Significant associations between driver gene mutations and DNA methylation alterations across many cancer types

**DOI:** 10.1371/journal.pcbi.1005840

**Published:** 2017-11-10

**Authors:** Yun-Ching Chen, Valer Gotea, Gennady Margolin, Laura Elnitski

**Affiliations:** Genomic Functional Analysis Section, National Human Genome Research Institute, National Institutes of Health, Bethesda, Maryland, United States; Sidney Kimmel Comprehensive Cancer Center, Johns Hopkins University, UNITED STATES

## Abstract

Recent evidence shows that mutations in several driver genes can cause aberrant methylation patterns, a hallmark of cancer. In light of these findings, we hypothesized that the landscapes of tumor genomes and epigenomes are tightly interconnected. We measured this relationship using principal component analyses and methylation-mutation associations applied at the nucleotide level and with respect to genome-wide trends. We found that a few mutated driver genes were associated with genome-wide patterns of aberrant hypomethylation or CpG island hypermethylation in specific cancer types. In addition, we identified associations between 737 mutated driver genes and site-specific methylation changes. Moreover, using these mutation-methylation associations, we were able to distinguish between two uterine and two thyroid cancer subtypes. The driver gene mutation–associated methylation differences between the thyroid cancer subtypes were linked to differential gene expression in JAK-STAT signaling, NADPH oxidation, and other cancer-related pathways. These results establish that driver gene mutations are associated with methylation alterations capable of shaping regulatory network functions. In addition, the methodology presented here can be used to subdivide tumors into more homogeneous subsets corresponding to underlying molecular characteristics, which could improve treatment efficacy.

## Author summary

Mutations that alter the function of driver genes by changing DNA nucleotides have been recognized as key players in cancer progression. However, recent evidence has shown that DNA methylation, which can control gene expression, is also highly dysregulated in cancer and contributes to carcinogenesis. Whether methylation alterations correspond to mutated driver genes in cancer remains unclear. In this study, we analyzed 4,302 tumors from 18 cancer types and demonstrated that driver gene mutations are inherently connected with the aberrant DNA methylation landscape in cancer. We showed that driver gene–associated methylation patterns can classify heterogeneous tumors within a cancer type into homogeneous subtypes and have the potential to influence genes that contribute to tumor growth. This finding could help us better understand the fundamental connection between driver gene mutations and DNA methylation alterations in cancer, and to further improve cancer treatment.

## Introduction

DNA methylation (DNAm) is highly dysregulated in cancers from many organs [1, 2], displaying aberrant CpG island (CGI) hypermethylation and long-range blocks of hypomethylation. Moreover, dysregulated DNAm at specific locations within the genome can often be used to divide heterogeneous tumors within cancer types into homogeneous subtypes [3, 4]. The origin of these dramatic changes in the DNAm of tumor cells remains a puzzle. On the one hand, DNAm alterations at particular CpG sites in tumors are associated with the aging process in normal cells [5, 6]. This has led some researchers to propose that cell proliferation, which drives age-associated DNAm errors in normal cells, is also responsible for aberrant DNAm in cancer [7]. On the other hand, although DNAm errors exhibit a linear association with the number of cell divisions in normal cells, they are not well correlated with the expression-based mitotic index in every type of cancer cell [7]. This suggests that in tumor cells, some factor other than cell proliferation is shaping the DNAm landscape. Because tumors of the same molecular subtype often harbor both dysregulated DNAm at particular locations in the genome and mutations in driver genes [3, 8], we decided to investigate the connection between somatic mutations and specific aberrant DNAm patterns.

Somatic mutations could directly or indirectly affect cancer methylomes. Frequent somatic mutations in epigenetic modifying enzymes could mechanistically explain dysregulated epigenomes, including DNA methylomes [9]. For example, mutations in *SETD2*, the H3K36me3 writer, lead to ectopic H3K36me3, coinciding with DNA hypermethylation in renal cell carcinomas [10]. In addition, mutations in driver genes whose functions do not directly influence the epigenome could result in downstream DNAm changes. For example, in glioblastoma, mutated *IDH1* produces abnormal 2-hydroxyglutarate. This leads to widespread CGI hypermethylation, termed the CpG island methylator phenotype (CIMP), by inhibiting the TET-demethylation pathway [11, 12]. In colorectal cancer, the BRAF V600E mutation results in DNA hypermethylation and CIMP development by upregulating the transcriptional repressor MAFG, which recruits the DNA methyltransferase DNMT3B to its targets at promoter CGIs [13]. Finally, the KRAS G13D mutation upregulates another transcriptional repressor, ZNF304, to establish a CIMP-intermediate pattern in colorectal cancer [14].

Somatic mutations and DNAm changes can co-occur in tumor molecular subtypes without clear mechanistic links. In head and neck squamous cell carcinomas (HNSCs), for instance, an atypical CIMP subtype was recently identified in association with *CASP8* mutations, which are not known to have a functional link to the epigenome [15]. And in gastric cancer, *PIK3CA* mutations co-occur with CIMP, which is thought to be caused by Epstein-Barr virus (EBV) infection [3]. In this type of cancer, *TP53* mutations are largely mutually exclusive with *PIK3CA* mutations. Thus, we would also expect *TP53* mutations to be associated with non-CIMP tumors. Despite lacking mechanistic links, it does not mean these non-random associations established simply by chance. Ascertaining these associations would be the critical first step for studying underlying mechanisms.

Based on these findings, we hypothesized that tumor genomic and epigenetic landscapes are stable and interdependent, and that specific driver mutations are associated with specific DNAm patterns. Thus, in this study, we systematically evaluated mutation-methylation associations across 4,302 tumors from 18 cancer types, along with 727 normal tissue samples from The Cancer Genome Atlas (TCGA). By investigating DNAm alterations associated with mutated driver genes on both a genome-wide scale and a site-specific scale, we were able to show that (i) mutated driver genes are tightly associated with DNAm variation in cancer; (ii) some driver gene associations are present across cancer types; for example, *TP53* mutations predominantly correspond to hypomethylation across cancer types; (iii) other associations are cancer type-specific; and (iv) these associations can be used to classify tumors into molecular subtypes and gain insight into functional alterations. Together, these results establish that driver mutations and DNAm alterations are tightly coupled in tumor cells, and that this coupling may affect important regulatory networks related to oncogenesis.

## Results

### Association between driver gene mutations and methylation patterns in cancer

**Fig 1:**
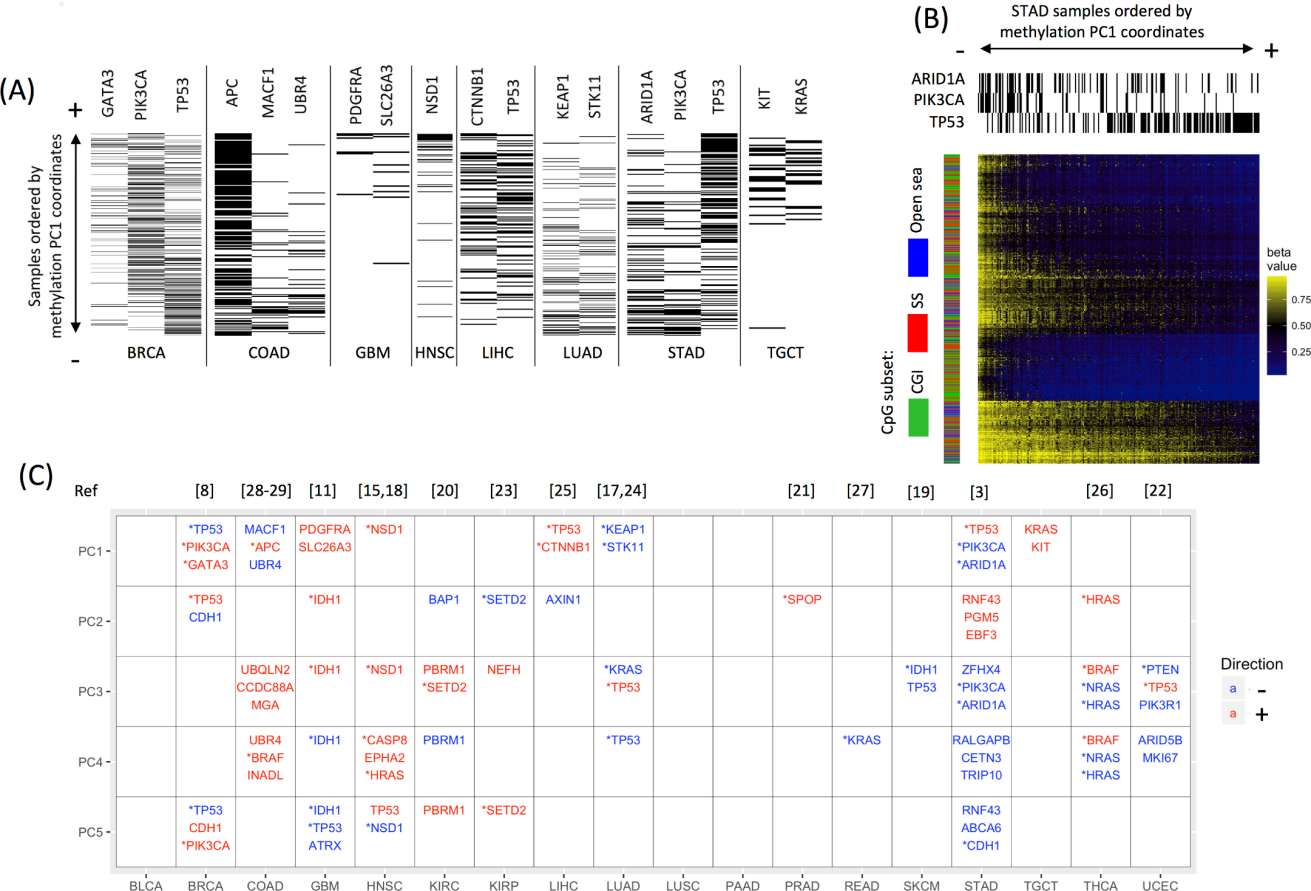
Driver gene mutations are significantly associated with DNA methylation in various cancers. (A) Examples of mutations in 15 driver genes that display an uneven distribution along the first principal component (PC1) of DNAm, biased toward either the positive extreme (+) or the negative extreme (-). Tumor samples are ordered vertically by their coordinates on PC1, from small (-, *bottom*) to large (+, *top*). A black line indicates the presence of the mutated driver gene in a sample, whereas a white line indicates its absence. Note that a sample’s presence at an extreme (+/-) of a PC does not necessarily correspond to high or low methylation. See Table 1 for cancer type abbreviations. (B) Example of a cancer with driver gene mutations unevenly distributed on PC1, resulting in distinct methylation patterns: *ARID1A/PIK3CA*-mutated stomach adenocarcinomas (STADs) display a methylation pattern distinct from *TP53*-mutated STADs. Shown is a heat map of methylation levels for the top 1,000 most heavily weighted probes in PC1. Each column represents a sample ordered by its PC1 coordinate, from small (-, *left*) to large (+, *right*). Each row represents a probe site. The three column sidebars on the top indicate mutation status for *TP53*, *PIK3CA*, and *ARID1A*. The row sidebar indicates the CpG subsets: CpG island (CGI), shores and shelves (SS; the 4-kb regions flanking the CGIs), or open sea (i.e., probes outside of CGIs and SSs). *TP53*-mutated STADs display lower methylation levels at the selected CpG sites than the majority of *ARID1A*/*PIK3CA*-mutated STADs. (C) In 15 of 18 cancer types examined, mutated driver genes were associated with one or more of the top five methylation PCs, shown as rows. The three driver genes most significantly associated with each PC are reported. Driver genes associated with the negative extreme of a PC are in blue, whereas associations with the positive extreme are in red. *Some of these mutated driver genes were previously reported in association with DNAm subtypes (corresponding references listed at the top).

To determine whether mutated driver genes were associated with methylation changes, first we performed principal component analysis (PCA) on methylation data for each of 18 different cancer types; within a given cancer type, tumor samples were projected onto the principal components (PCs). Illumina Infinium human methylation 450K array data and somatic mutation data were downloaded from TCGA (Table 1), and driver genes were predicted with MutSigCV [16] (see Materials and Methods for details). For each cancer type, a driver gene was considered to be associated with a PC if samples in which the gene was mutated (any synonymous/non-synonymous mutation reported in TCGA level 2 exome-sequencing data) were unevenly distributed toward the positive or negative extremes of that PC (q<0.05; two-sided Wilcoxon rank-sum test). We assessed each driver gene for the top five DNAm PCs; examples of PC1-associated driver genes are shown in Fig 1A.

**Table 1:** Number of tumor samples, normal samples^a^, and driver genes across 18 cancer types.

Cancer type	Abbr.	# tumor samples	# normal samples	# driver genes^b^
Bladder urothelial carcinoma	BLCA	130	21	48
Breast invasive carcinoma	BRCA	652	102	51
Colon adenocarcinoma	COAD	219	38	650
Glioblastoma multiforme	GBM	144	2	16
Head and neck squamous cell carcinoma	HNSC	306	60	34
Kidney renal clear cell carcinoma	KIRC	245	160	14
Kidney renal papillary cell carcinoma	KIRP	153	45	10
Liver hepatocellular carcinoma	LIHC	202	50	11
Lung adenocarcinoma	LUAD	407	32	84
Lung squamous cell carcinoma	LUSC	74	43	10
Pancreatic adenocarcinoma	PAAD	90	10	31
Prostate adenocarcinoma	PRAD	261	50	22
Rectum adenocarcinoma	READ	80	7	7
Skin cutaneous melanoma	SKCM	372	3	56
Stomach adenocarcinoma	STAD	244	2	366
Testicular germ cell tumor	TGCT	142	0	25
Thyroid carcinoma	THCA	446	56	5
Uterine corpus endometrial carcinoma	UCEC	135	46	62
**Total**		4,302	727	1,502

^a^Normal tissue samples were obtained from tissues adjacent to tumors. Abbr. = TCGA abbreviated nomenclature

^b^Defined as MutSigCV-reported driver genes mutated in at least five tumor samples for a given cancer type in our data set (see MutSigCV in [16]).

A PC-associated driver gene suggests that the mutated samples at one extreme of the PC display methylation patterns distinct from the non-mutated samples at the other extreme. For instance, in stomach adenocarcinoma (STAD), *TP53*-mutated samples were distributed toward the positive extreme of PC1, whereas *ARID1A*- and *PIK3CA*-mutated samples were distributed toward the negative extreme. Thus, distinct methylation patterns associated with PC1 separated the majority of *TP53*-mutated STAD samples from *ARID1A*- and *PIK3CA*-mutated samples: *ARID1A*- and *PIK3CA*-mutated samples were highly methylated at PC1-defining probes relative to *TP53*-mutated samples (Fig 1B). Overall, we found a significant association between 159 driver genes and one or more of the top five methylation PCs in 15 of 18 cancer types. The top three driver genes associated with each PC are shown in Fig 1C; some have been previously reported in association with DNAm subtypes [3, 8, 11, 15, 17–29] (see Fig 1C for the references associated with each gene). For each cancer type, the full list of driver genes associated with methylation PCs can be found in S1 Table, and the full list of MutSigCV-reported driver genes can be found in S2 Table.

Mathematically, distinct PCs represent mutually orthogonal (uncorrelated) linear combinations of probes corresponding to different methylation patterns (such as the pattern associated with PC1 in STAD, shown in Fig 1B). Several top PCs usually capture the majority of variance in the methylome. Thus, the frequent driver gene–PC associations in almost every cancer type suggest a tight connection between driver gene mutations and DNA methylation alterations in cancer.

Next, we investigated whether the mutation-methylation connection in cancer was limited to certain CpG subsets—namely those occurring in CGIs, shores and shelves (SSs; the 4-kb regions flanking the CGIs), or open sea regions (i.e., probes outside of CGIs and SSs)—as the regulatory functions of these CpG subsets may differ [30]. For example, DNAm in promoter CGIs often causes gene silencing. DNAm in CGI shores is frequently altered and strongly correlated to corresponding gene expression in cancer [31]. And in addition to containing repetitive elements, open sea regions also contain functional sites such as enhancers, exons, and introns, where DNAm changes may affect gene regulation and splicing. Thus, we repeated the analysis described above for each subset of probes. We observed similar driver gene–PC associations across multiple cancer types, indicating that the mutation-methylation connection is not limited to a particular CpG subset (S1 Fig). In total, 14 of 18 cancer types harbored significant associations between driver gene mutations and the top five methylation PCs at CGIs, 14 of 18 at SSs, and 15 of 18 in open sea regions. We then repeated the same analysis after stratifying probes by hypo- or hypermethylation status and found that the results did not vary appreciably (S1 Fig). Of note, in this study we identified hyper- and hypomethylated probes by comparing the methylation of tumor and normal samples (q<0.05; Wilcoxon rank-sum test).

Some researchers have recently proposed that aberrant DNAm in cancer is driven by cell proliferation and developed a DNAm-based mitotic index (derived from the average methylation level across 385 CpG sites) [7]. We found that the top 5 PCs correlated with the DNAm-based mitotic index in all cancer types (S2 Fig), suggesting that mutated driver genes, DNAm patterns, and cell proliferation rates are associated (see S1 Text for detailed information). However, after removing probes correlated with the DNAm-based mitotic index (p<0.05; Pearson correlation), methylation PC–driver gene associations remained for 11 cancer types (S3 Fig), indicating that mutation-methylation associations cannot be totally explained by the DNAm-based mitotic index. In addition, we found that the findings for the DNAm-based mitotic index were not consistent with those for an expression-based mitotic index (derived from the average expression level across 9 mitotic genes) in association with some methylation PCs (S2 Fig) and driver gene mutations (S3 Table). For example, *TP53*-mutated tumors are associated with a high expression-based mitotic index in 9 cancer types, whereas no such association was seen for the DNAm-based mitotic index in any cancer type. This inconsistency points out further investigation is needed to elucidate the relationship among cell proliferation, driver gene mutations, and DNAm variation.

### Driver gene mutations, genome-wide CGI hypermethylation, and open sea hypomethylation in tumors

We next asked whether driver gene–associated methylation alterations correspond to genome-wide methylation patterns characteristic of cancer: i.e., widespread CGI hypermethylation and huge hypomethylated blocks, primarily in open sea regions [1, 2, 32]. To answer this question, we calculated HyperZ and HypoZ indices for each sample [33]. A high HyperZ index indicates that aberrant hypermethylation exists in many CGIs for a given sample, whereas a high HypoZ index indicates that extensive open sea hypomethylation is present. Mutated driver genes that were significantly associated with either high or low HyperZ and/or HypoZ indices are shown for all 18 cancer types in Table 2 (q<0.05; Wilcoxon rank-sum test); the number of associated driver genes varies from 67 in COAD to 0 in pancreatic adenocarcinoma (PAAD), rectum adenocarcinoma (READ), and skin cutaneous melanoma (SKCM).

**Table 2:** Associations between mutated driver genes and HyperZ and HypoZ indices or site–specific methylation alterations.

Cancer type	Driver genes associated with HyperZ and HypoZ (in alphabetical order)^a^			# driver genes associated with any probe^b^
	**HyperZ**	**HypoZ**	**Total**	
**BLCA**	**STAG2**	NA	1	0
**BRCA**	FOXA1	**CDH1**	2	50
**COAD**	ADORA3,AKAP9,ARAP3,ATG2B, BCL9,BRAF,BRD8,CAB39L,CCDC88A, CD58,CDK12,CEL,CHD3,CYLC1, DAXX,DNAH17,DOCK1,DSG4,EGR1, ERCC3,FBN2,FHOD3,FMN2,GIGYF2, GPATCH8,GPR112,HEATR4,HIPK2, HIST1H1E,IGF2R,INADL,IQSEC2, KAT2B,KBTBD4,KCTD3,KLKB1, LATS1,LEPRE1,MACF1,MBD6,MGA, MTA2,MTTP,NOVA1,OR4M2,OVCH1, PCDHGA11,PLAGL2,PLEKHA6, PLXNA3,PREX2,PSD,PTCH1,RNF43, SPEN,SVIL,TET3,TNRC6C,UBQLN2, UBR4,USP34,XYLT2,ZBTB20,ZFYVE26	**C14orf39**,**CNTNAP5**,**FBN2**, **MEPE**,**SPEN**	67	189
**GBM**	IDH1,**PDGFRA**,**STAG2**	PDGFRA,STAG2	3	16
**HNSC**	CASP8	NSD1	2	34
**KIRC**	BAP1,PBRM1,SETD2	ARID1A	4	14
**KIRP**	NF2,SETD2		2	10
**LIHC**		CTNNB1,TP53	2	11
**LUAD**	**STK11**	KEAP1,LTBP1,STK11,TP53	4	80
**LUSC**		TP53	1	0
**PAAD**			0	7
**PRAD**	SPOP	TP53	2	12
**READ**			0	0
**SKCM**			0	43
**STAD**	ADNP2,ALG10,ARID1A,BCL9L,BCOR, C5orf42,EIF5B,EPHA2,FHOD3,GLI1, GON4L,IRS4,KIAA0195,PFKP,PIK3CA, RALGAPB,RNF43,STAB1,TBX4,TLE2, **TP53**,TP53BP2,WASF3,WDR7,WNT16, XYLT2,ZBTB20,ZFHX4,ZNF608	FRMD4A,TP53,ZFHX4	30	357
**TGCT**	NA^c^	NA^c^	NA^c^	25
**THCA**	**BRAF**,HRAS,NRAS	BRAF,**EIF1AX**,**HRAS**,**NRAS**	4	5
**UCEC**	CUX1,PIK3R1,PTEN	TP53	4	53

^a^ Negatively associated genes are shown in bold; positively associated genes are shown in normal font.

^b^ See S2 Table for driver genes associated with site-specific methylation alterations

^c^ Normal tissue samples were not available to calculate HyperZ/HypoZ indices

Some known players in oncogenesis appear on this list. For example, a high HyperZ index was associated with *BRAF* in COAD and *IDH1* in glioblastoma (GBM); both genes are linked to CIMP in cancer [11, 29]. And *NSD1* (which encodes a histone methyltransferase) was associated with a high HypoZ index in HNSC and has also been linked to hypomethylation in cancer [18]. The associations we detected in most cancer types underscore the relationship between driver gene mutations and the genome-wide methylation alterations commonly observed in cancer. Only a few cancer types lacked these associations.

### Driver gene mutations and site-specific methylation alterations in tumors

Next, we investigated whether the connection between driver gene mutations and methylation alterations was methylation site–specific in each cancer type. To do so, we calculated the associations between every driver gene and every methylation array probe for all 18 cancer types, testing whether the presence of mutations in a driver gene was associated with high or low methylation levels at a given probe site (q<0.05; Wilcoxon rank-sum test). Across almost all cancer types, many more driver genes were significantly associated with at least one probe than with the HyperZ and/or HypoZ indices, after correcting for multiple testing (Table 2). In total, 737 unique driver genes were implicated, and driver gene–methylation site associations were present genome-wide. An example of the chromosomal distribution of driver gene–associated methylation probes present in kidney renal clear cell carcinoma (KIRC) is shown in S4A Fig. The numerous gene-probe associations detected in KIRC suggest that driver gene–associated methylation changes likely occur at certain CpG sites, potentially resulting from a site-targeting mechanism. A heat map illustrates mutations in these 14 genes in KIRC (S4B Fig), showing some co-occurrence between *SETD2* mutations and *PBRM1* mutations, and between *BAP1* mutations and *PBRM1* mutations, whereas *SETD2* mutations and *BAP1* mutations are almost mutually exclusive.

**Fig 2:**
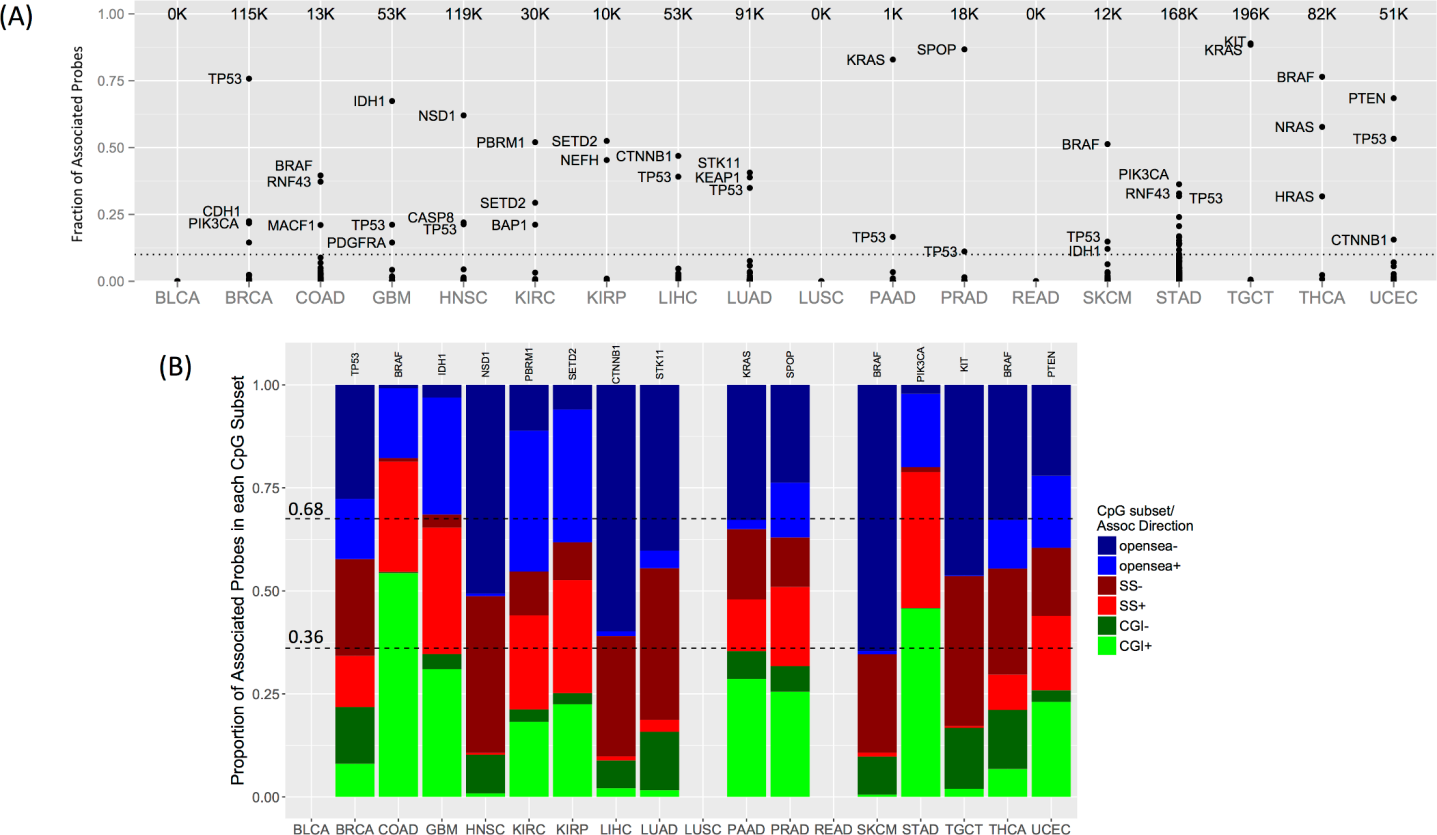
Driver gene–methylation associations and CpG subsets. (A) The total number of probes associated with any driver gene is shown for each cancer type (*top of each column*). Each point represents the fraction of corresponding probes associated with a driver gene (y-axis). Names are shown for each of the top three driver genes if they account for more than 10% of total probes (*dotted line*). See Table 1 for cancer type abbreviations. (B) Driver genes with the most probe associations in each cancer type (*gene names in panel*). The bar plots show the proportion of associated probes in each of the three CpG subsets [CpG islands (CGIs), shores and shelves (SSs), or open sea], stratified by the direction of association (+/-). Dashed lines indicate the divisions expected if associations were proportionally distributed. No probes were associated with driver genes in BLCA, LUSC, and READ.

The number of probes associated with each driver gene varied greatly, ranging from fewer than 10 to tens of thousands (S2 Table). For each cancer type, a few (1–5) dominant driver genes accounted for the majority of associations (Fig 2A). These dominant driver genes included known oncogenes and tumor suppressor genes such as *TP53*, *PTEN*, and *PIK3CA*, and known CIMP-driving genes such as *BRAF*, *IDH1*, and *KRAS* [13, 14, 34]. Dominant driver genes usually displayed both positive and negative associations with probe methylation levels in a given cancer type (Fig 2B). However, there was typically more of one type of association than the other. By definition, positive associations indicate higher methylation levels among tumor samples in the presence of driver gene mutations, whereas negative associations indicate lower methylation levels. Thus, positive associations would correspond to hypermethylation (primarily in CGIs) if normal samples displayed low methylation levels at a given probe site, whereas negative associations would correspond to hypomethylation (primarily in open sea regions) if normal samples displayed high methylation levels.

Primarily positive associations occurred for the CIMP-driving genes *BRAF* in COAD and *IDH1* in GBM. Other genes with predominantly positive associations included *RNF43* and *MACF1* in COAD; *CASP8* in HNSC; *PBRM1* (a chromatin remodeler), *SETD2* (a histone methyltransferase), and *BAP1* (a histone deubiquitinating enzyme) in KIRC; *SETD2* in kidney renal papillary cell carcinoma (KIRP); *SPOP* in prostate adenocarcinoma (PRAD); *PIK3CA* in STAD; and *PTEN* in uterine corpus endometrial carcinoma (UCEC). These genes were also associated with a high HyperZ index, suggesting that they may play a role in genome-wide CGI hypermethylation in particular cancer types (Table 3).

**Table 3:** Mutated driver genes that exhibit primarily positive or negative probe associations and correspond to a high HyperZ or HypoZ index in particular cancer types.

Gene	Cancer type	P-Assoc^a^	N-Assoc^a^	HyperZ	HypoZ
BRAF	COAD	5166	103	Y	
RNF43	COAD	4784	173	Y	
MACF1	COAD	2706	95	Y	
IDH1	GBM	31877	3535	Y	
CASP8	HNSC	21552	4660	Y	
PBRM1	KIRC	11831	3906	Y	
SETD2	KIRC	6443	2440	Y	
BAP1	KIRC	5162	1240	Y	
SETD2	KIRP	4300	936	Y	
SPOP	PRAD	8919	6444	Y	
PIK3CA	STAD	58843	2153	Y	
PTEN	UCEC	20548	14496	Y	
NSD1	HNSC	1423	72475		Y
CTNNB1	LIHC	1065	23869		Y
STK11	LUAD	3269	33840		Y
KEAP1	LUAD	4122	31348		Y
RNF43	STAD	31905	23267	Y	Y

^a^ Number of positively (P-Assoc) and negatively (N-Assoc) associated probes are reported for each driver gene in particular cancer types.

By contrast, genes that primarily displayed negative associations were often associated with a high HypoZ index, suggesting that they may play a role in genome-wide open sea hypomethylation. Examples were *NSD1* in HNSC, *CTNNB1* in liver hepatocellular carcinoma (LIHC), and *STK11* and *KEAP1* in lung adenocarcinoma (LUAD) (Table 3). Interestingly, *RNF43* was associated with both high HyperZ and HypoZ indices in STAD, suggesting a dual role in genome-wide CGI hypermethylation and open sea hypomethylation. Genomic distribution analysis on *RNF43*-associated probes revealed that positively associated probes were enriched in gene promoters, whereas negatively associated probes were enriched in gene bodies, suggesting that they may have different functional impacts (S5 Fig).

In short, a few driver genes were linked to genome-wide patterns of CGI hypermethylation and open sea hypomethylation in particular cancer types, whereas many more driver genes were linked to a few probe sites aberrantly methylated in cancer (S2 Table). We note that no probe-level associations were identified for bladder urothelial carcinoma (BLCA), lung squamous cell carcinoma (LUSC), and READ. However, this lack of significant associations could reflect the relatively small sample size for these cancers. When we re-analyzed the data by combining COAD and READ, all mutated driver genes in READ were associated with probes in the combined set and more gene-probe associations were seen in the combined set than in COAD alone (results for the combined set shown in the ‘CRAD’ tab in S2 Table).

### Consistency of methylation alterations associated with mutated driver genes across cancer types

**Fig 3:**
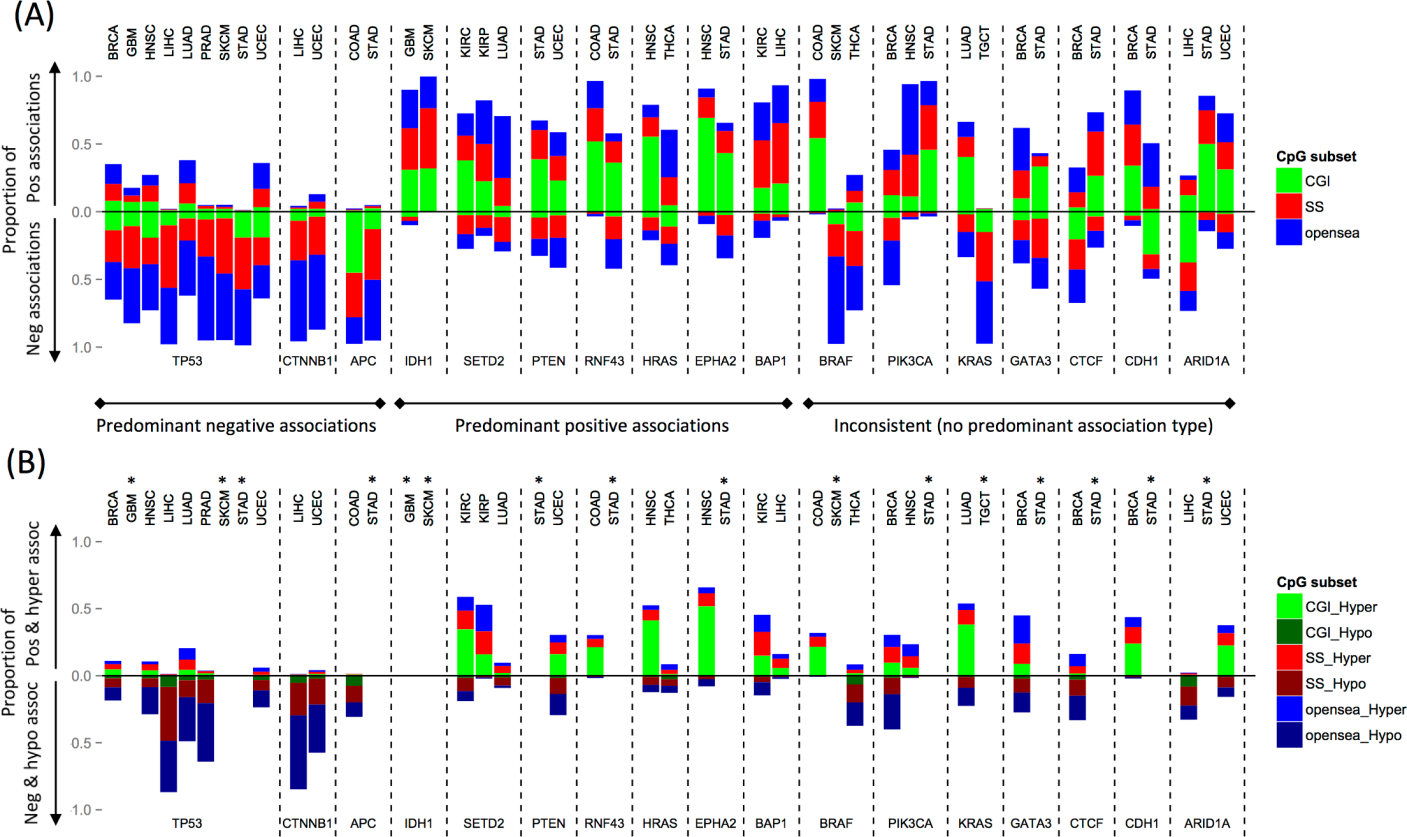
Proportion of positive and negative associations with methylation for 17 recurrently mutated driver genes. (A) Bar plots show the proportion of methylation probes for each driver gene (*labels at bottom*) and cancer type (*labels at top*) displaying positive and negative associations. Positive associations are plotted above the horizontal line, negative associations below the horizontal line. Associations are further stratified by CpG subset: CpG islands (CGI), shores and shelves (SS), and open sea (regions outside CGIs and SSs). Driver genes were classified into three groups based on the directionality of their predominant associations (*negative*, *positive*, *inconsistent*). All genes shown were associated with more than 1,000 probes, in at least two cancer types. See Table 1 for cancer type abbreviations. (B) Plotted as in (A), using: (1) positively associated and hypermethylated probes and (2) negatively associated and hypomethylated probes. *Hyper- or hypomethylated probes were not identified for glioblastoma (GBM), stomach adenocarcinoma (STAD), skin cutaneous melanoma (SKCM), and testicular germ cell tumor (TGCT) due to a lack of normal samples.

Next, we asked whether mutated driver genes consistently displayed predominantly positive/negative associations across multiple cancer types. We investigated the proportion of positive and negative probe associations for 17 driver genes across 18 cancer types (Fig 3A). These genes were selected because they were associated with extensive methylation alterations (more than 1,000 probe associations per driver gene) in at least two cancer types. When compared with normal samples, positive associations often equated to hypermethylation (primarily in CGIs) in response to mutations. Likewise, negative associations often equated to hypomethylation (primarily in open sea regions) (Fig 3B).

*TP53* displayed predominantly negative associations in 9 of 18 cancer types, and no predominantly positive associations were observed in connection to this gene in any cancer type. This suggests a tight connection between *TP53* mutations and open sea hypomethylation across multiple cancer types. Interestingly, many negatively associated probes were shared across cancer types (62,951 probes were shared across 2 cancer types, and 15 were shared across 7 types) (S6 Fig). *APC* and *CTNNB1* also displayed predominantly negative associations in two different cancer types each.

By contrast, *IDH1* strongly favored positive associations in two cancer types, GBM and SKCM, consistent with reports that mutated *IDH1* downregulates TET-dependent demethylation, resulting in aberrant CGI hypermethylation [35]. *SETD2*, *PTEN*, *RNF43*, *HRAS*, *EPHA2*, and *BAP1* were also linked to primarily positive associations in more than one cancer type, suggesting that they may play a general role in CGI hypermethylation.

Finally, *BRAF*, which mediates CIMP in colorectal cancer, displayed a high proportion of positive associations (0.98) in COAD, but low proportions in SKCM (0.02) and thyroid carcinoma (THCA; 0.27). Its negative associations in THCA corresponded to hypomethylation across CGIs, SSs, and open sea regions (Fig 3B and S2 Table). This dramatic difference indicates that driver genes may be associated with methylation patterns in a cancer type–specific manner. Such cancer type–specific associations were also seen for *PIK3CA*, *KRAS*, *GATA3*, *CTCF*, *CDH1*, and *ARIDA1*.

### Functional implications of *TP53*-associated probes across cancer types

The consistent predominantly negative trend in *TP53*-methylation associations across cancer types led us to ask whether *TP53*-associated methylation alterations might functionally converge along the same biological pathways. To focus solely on *TP53* mutations, first, we recomputed *TP53*-associated probes by performing multiple testing correction for *TP53*-probe associations within each cancer type without considering other driver gene-probe associations (q<0.05). *TP53*-associated probes were seen in 11 cancer types with the number varying from 2,940 (PAAD) to 141,002 (BRCA) (S4 Table). Negative associations predominated in all cancer types, consistent with the previous analysis. For each cancer type, we then identified genes whose expression levels were correlated with *TP53*-associated probes (q<0.05; Spearman correlation) in gene promoters or bodies exhibiting aberrant methylation changes (magnitude of median difference in beta values between *TP53*-mutated tumors and normal samples >0.1). The correlated genes were further stratified into up- and downregulated genes in *TP53*-mutated tumors, relative to both tumors without *TP53* mutations and normal samples (median difference in expression level > 0.5; log2 RSEM). To test whether the aberrantly regulated genes converged on common pathways, we performed gene set analysis for genes that were up- or downregulated in at least two cancer types. On the one hand, downregulated genes were enriched in 25 regulatory gene sets (q<0.05), with the strongest corresponding to FOXO4 targets (q = 6E-5). No enriched canonical pathways were found (S5 Table). On the other hand, upregulated genes were enriched in 38 canonical pathways, with the strongest enrichment present in the cell cycle/mitotic pathway (q = 3.5E-15, hypergeometric test) and 21 regulatory gene sets mainly corresponding to E2F targets (q<0.05) (S5 Table). The enriched pathways/gene sets remained largely the same when repeating the analysis restricted to genes corresponding to *TP53*-negatively associated probes, whereas no enriched pathways/gene sets were found for genes corresponding to *TP53*-positively associated probes. This implies that, across cancer types, the enrichment was dominated by genes corresponding to the probe sites exhibiting lower methylation levels in *TP53*-mutated tumors.

### Identification of molecular subtypes in thyroid carcinoma and uterine corpus endometrial carcinoma based on driver gene–associated methylation patterns

In previous studies, researchers have identified cancer subtypes in COAD and GBM by matching mutational profiles to methylation patterns [11, 29]; here, we asked whether site-specific mutation-methylation associations could separate tumors into subtypes. We focused on the methylation patterns associated with the top three driver genes in THCA and UCEC, which account for the most probe associations (as shown in Fig 2A), because in these cancers the top three genes rarely co-occurred. Thus, if subtypes linked to mutation-methylation associations were present, they would probably display a clear separation. In THCA, the top three genes (*NRAS*, *HRAS*, or *BRAF*) were each mutated in a mutually exclusive fashion. And in UCEC, mutations in *TP53* were nearly mutually exclusive with *PTEN* and *CTNNB1* mutations, which co-occurred in many tumor samples.

**Fig 4:**
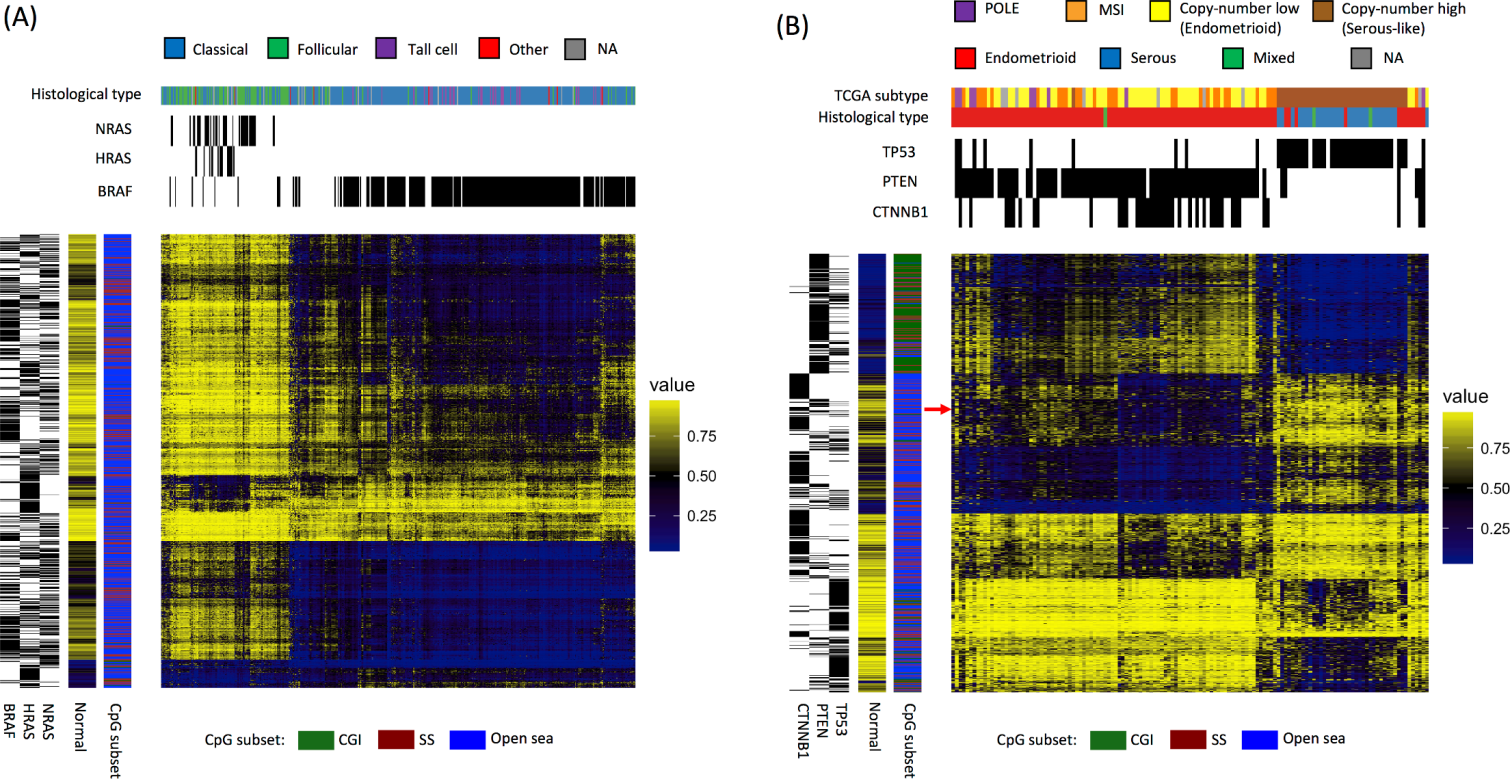
Driver gene–associated methylation patterns can be used to identify tumor molecular subtypes. Heat maps for (A) thyroid carcinoma (THCA) and (B) uterine corpus endometrial carcinoma (UCEC) depict hierarchical clustering of methylation values of the union set of the 500 probes most significantly associated with each of the three dominant driver genes in each cancer type. Each column represents a sample, and each row represents a probe. Mutation status and subtype classification are shown in the upper sidebars. The sidebars on the left indicate gene-probe associations and CpG subsets, as well as average methylation levels across normal samples. The arrow in (B) indicates methylation probes that display more hypomethylation in samples where *PTEN* and *CTNNB1* mutations co-occur than in samples with *PTEN* mutations alone.

For both cancer types, we performed hierarchical clustering on the union of the 500 methylation probes most significantly associated with mutations in each of the top three genes (Fig 4). In THCA, two methylation subtypes emerged, corresponding to *NRAS*- and *HRAS*-mutated tumors vs. *BRAF*-mutated tumors. These two groups were consistent with the follicular vs. classical histological subtypes of THCA, respectively (Fig 4A). The selected probes primarily fell in non-CGI positions (i.e. SS and open sea regions); *BRAF* mutants displayed hypomethylation in open sea and some SS regions, whereas *NRAS* and *HRAS* mutants displayed methylation levels similar to normal samples in open sea and SS regions, with little hypermethylation. Furthermore, we found tumors lacking any of the specified mutations that co-clustered within these patterns. No evidence of increased expression or copy number gain in *BRAF* or *NRAS/HRAS*, or of significant enrichment of mutated genes other than *BRAF*, *NRAS*, and *HRAS* were found in these co-clustered tumors. This suggests either that unknown events drive these co-clustered tumors toward similar molecular profiles, or that *NRAS*, *HRAS* or *BRAF* mutations have been missed in these tumors.

Two methylation subtypes were also identified in UCEC, this time corresponding to *TP53* vs. *PTEN* mutations, consistent with the serous vs. endometrioid histological subtypes of UCEC, respectively (Fig 4B). *PTEN-*mutated samples generally exhibited CGI hypermethylation, whereas *TP53*-mutated samples generally exhibited normal methylation levels, with some hypomethylation in open sea regions. Most UCEC samples with mutations in both *PTEN* and *CTNNB1* displayed greater levels of open sea hypomethylation than samples with *PTEN* mutations alone, a finding which has not been previously reported. Moreover, these samples corresponded to copy-number low subtype according to TCGA classification (Fig 4B).

Collectively, these results illustrate the connectivity between mutational profiles and DNA methylation in cancer. We note that the same methylation subtypes in both cancer types can be found by using the top 1% most variable probes as well (S7 Fig).

### Correlations between driver gene–associated methylation probe sites and corresponding gene expression in two thyroid carcinoma subtypes

Finally, we investigated whether driver gene–associated methylation patterns shape gene regulatory networks. To investigate the three-way association between mutation, methylation, and gene expression, we used THCA as our primary example; the mutually exclusive mutation profile present in this type of cancer minimized the complexity of the associated methylation patterns, facilitating the study of gene expression. We looked for genes whose aberrant expression levels were correlated with aberrant methylation levels (each relative to normal samples). We focused on CpG sites in promoter regions and gene bodies in *NRAS* and *HRAS* mutants (the *NRAS-HRAS* group) vs. *BRAF* mutants (the *BRAF* group). These genes were subsequently sorted into four different categories based on expression: (1) upregulation only in the *BRAF* group, (2) upregulation only in the *NRAS*-*HRAS* group, (3) downregulation only in the *BRAF* group, and (4) downregulation only in the *NRAS*-*HRAS* group. For each category, we reported the affected genes, sorted by median difference in expression between mutants and normal samples (S6 Table), and significantly enriched pathways (S7 Table).

**Fig 5:**
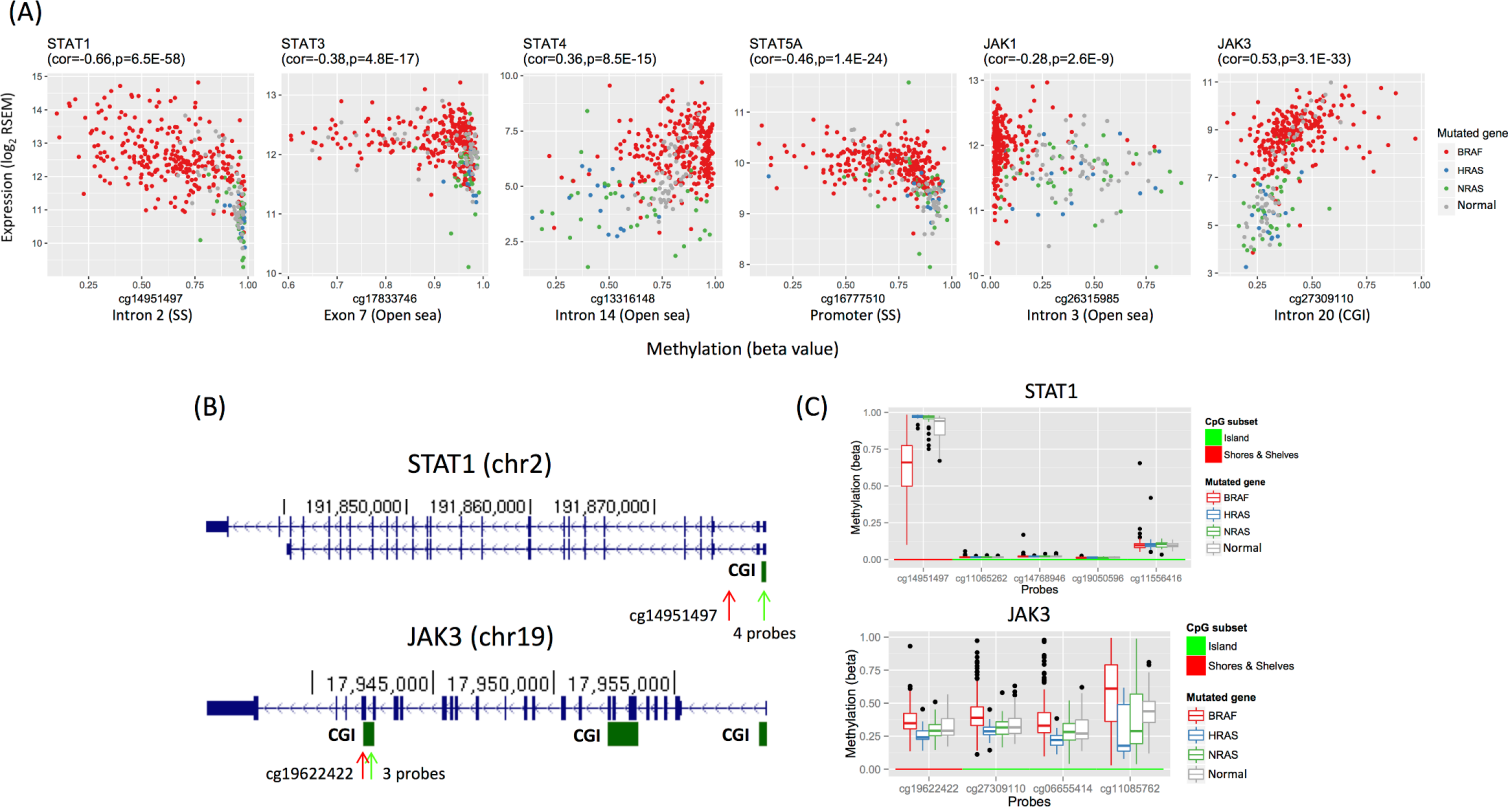
Differential expression of JAK and STAT family genes is correlated with differential methylation in thyroid cancer subtypes. (A) Shown are scatter plots for gene expression levels (y-axis) and methylation levels (x-axis) for *STAT1*, *STAT3*, *STAT4*, *STAT5A*, *JAK1*, and *JAK3*, plotted with *BRAF*-mutated (*red*), *HRAS*-mutated (*blue*), *NRAS*-mutated (*green*), and normal (*grey*) samples. Gene names and Spearman rho values (with p-values) for the correlation between gene expression and methylation among tumor samples are shown on top of the plots. Probe names (where methylation levels were measured) and genomic locations are shown on the bottom of the plots. (B) Snapshots from the UCSC genome browser for *STAT1* and *JAK3*, with CpG islands (CGIs) indicated below (*green arrow*: CGIs; *red arrow*: probes flanking the CGIs). Methylation levels of the indicated regions are shown in panel (C). (C) Box plots show methylation levels (y-axis) at probes in *STAT1* and *JAK3* for *BRAF*-mutated, *HRAS*-mutated, *NRAS*-mutated, and normal samples. The shown probes fall in the north shores and shelves [or SSs, indicated by red arrows in (B)] of the *STAT1* promoter CGI and the 3’ CGI of *JAK3* [indicated by green arrows in (B)].

In all, 1,565 differentially methylated genes were upregulated specifically in the *BRAF* group (S6 Table). Gene set analysis showed that these genes were enriched in 97 canonical pathways, which were primarily pertinent to cell-cell communication/extracellular matrix gene sets and signaling pathways (q<0.05; hypergeometric test; S7 Table). Some upregulated genes were involved in many of the 97 pathways; the 10 genes implicated in the most pathways were: *GRB2* (present in 34 out of 97 pathways), *RAC1* (*n* = 26), *STAT1* (*n* = 25), *LYN* (*n* = 24), *VAV1* (*n* = 24), *JAK1* (*n* = 22), *PTPN6* (*n* = 22), *ITGB1* (*n* = 20), *STAT3* (*n* = 18), and *STAT5A* (*n* = 18). We noticed that 4 genes (*STAT1*, *JAK1*, *STAT3*, and *STAT5A*) corresponded to the JAK and STAT gene families implicated in many signaling cascades, including the KEGG JAK-STAT signaling pathway (ranked 12^th^ out of the 97 pathways; q = 4.7E-5). In addition, *JAK3* (*n* = 11) and *STAT4* (*n* = 4), other two members in JAK and STAT gene families, were also upregulated in multiple pathways. This differential regulation of the JAK and STAT families may be shaped by differences in DNA methylation (Fig 5A). Specifically, *STAT1* differential expression is negatively correlated with methylation levels at an SS probe of the promoter CGI, whereas *JAK3* differential expression is positively correlated with methylation levels at the 3’ gene body CGI and its north shore (Fig 5B and 5C). Finally, 9 of the top 15 differentially methylated genes were upregulated in the *BRAF* group and involved in metastasis: *KLK6*, *KLK7*, *KLK11* [36], *CLDN10* [37], *B3GNT3* [38], *RASGRF1* [39], *ST6GALNAC5* [40], *TACSTD2* (also known as *TROP2*) [41], and *CEACAM6* [42] (S6 Table).

By contrast, 1,043 differentially methylated genes were downregulated specifically in the *BRAF* group. Gene set analysis showed that these genes were enriched in five canonical pathways, which were pertinent to amino acid catabolism, triglyceride biosynthesis, glycerophospholipid metabolism, and nucleic acid metabolism (S7 Table).

The *NRAS-HRAS* group displayed 278 differentially methylated, upregulated genes. These genes were enriched in three canonical pathways, which were relevant to the neuronal system, potassium channels, and melanogenesis (S7 Table). We did not find a substantial proportion of differentially methylated genes implicated in tumor progression among the top differentially expressed genes (defined by median difference in expression between *NRAS-HRAS* mutants and normal samples). However, when we considered the top 17 differentially expressed, highly transcribed genes (median expression level in mutants > 10 log2 RSEM; median difference > 1 log2 RSEM; highlighted in S6 Table in bold), 6 out of 17 were implicated in tumorigenesis. These genes included G protein alpha subunit (*GNAS*) [43], pyruvate dehydrogenase kinase 4 (*PDK4*) [44], NADPH reductase (*NQO1*) [45], and three NADPH oxidases that produce H_2_O_2_ for thyroid hormone synthesis: *DUOX2*, *DUOXA2*, and *DUOX1* [46] (S6 Table).

Finally, 447 differentially methylated genes were downregulated specifically in the *NRAS-HRAS* group. These genes were enriched in 166 canonical pathways; interestingly, 47 genes overlapped the 97 pathways enriched in the *BRAF* upregulated group, including the JAK-STAT signaling pathway (ranked 13^th^, *q* = 3.5E-5). Specifically, *STAT1*, *STAT3*, *STAT4*, and *JAK3* were among the genes upregulated in the *BRAF* group but downregulated in the *NRAS-HRAS* group (Fig 5A and S6 Table). This result demonstrates that methylation changes are indeed associated with differential gene expression between *BRAF*-mutated and *NRAS*- and *HRAS*-mutated samples in THCA.

## Discussion

In this study, we demonstrated that driver gene mutations are tightly tied to the DNAm landscape in multiple types of cancer. Furthermore, we showed that mutated driver genes are associated with DNAm alterations in a reproducible, site-specific manner. In each cancer type, a few driver genes dominate the site-specific associations, and some potentially contribute to CGI hypermethylation and extensive hypomethylation, i.e., the hallmarks of cancer. We caution that these findings do not equate to causality, but do point to the highly interconnected nature of the genome and epigenome.

Our findings are consistent with previous research on methylation in cancer; however, they also contribute novel insights. Several driver genes that displayed primarily positive or negative associations with methylation probes in this study have been previously linked to CGI hypermethylation or open sea hypomethylation, respectively. Driver genes associated with CGI hypermethylation in both this study and past studies include *BRAF* in COAD [29], *IDH1* in GBM [11], *SETD2* in KIRC [10], *PIK3CA* in STAD [3], *CASP8* in HNSC [15], *SPOP* in PRAD [21], and *PTEN* in UCEC [47]. Genes associated with hypomethylation include *TP53* in LIHC [48], *BRAF* in THCA [26], and *NSD1* in HNSC [18]. In addition to these examples, we identified novel driver genes that may contribute to CGI hypermethylation, such as *BAP1* in KIRC, or to open sea hypomethylation, such as *CTNNB1* in LIHC. By illuminating the driver genes associated with widespread DNAm alterations, as well as driver genes associated with more limited DNAm alterations, our comprehensive analysis provides a detailed mutation-methylation map for many types of cancer.

Several mutated driver genes displayed consistent and widespread positive or negative associations across cancers, corresponding to extensive DNAm alterations. The effects of others varied by cancer type. This discrepancy may be attributable to different underlying mechanisms. For example, mutations in *IDH1* and *SETD2* directly affect the epigenetic landscape by inhibiting TET-dependent demethylation and disturbing DNA methyltransferase targeting, respectively [10, 12, 35]. Both mechanisms cause DNA hypermethylation, in line with the corresponding primarily positive associations observed in this study. *BRAF* mutations, by contrast, displayed inconsistent methylation patterns between cancer types in this study. In COAD, *BRAF*-mutated samples mutations displayed widespread CGI hypermethylation. This is consistent with our knowledge that, in this type of cancer, BRAF V600E recruits DNA methyltransferase to CGI targets by stimulating the MEK/ERK signaling pathway and upregulating the transcription repressor MAFG [13]. However, in THCA, *BRAF*-mutated samples (260/266 of which harbored the V600E mutation) largely displayed hypomethylation. Although no mechanistic explanation for this observation is yet available, it is possible that the mutation does not upregulate MAFG in THCA. Alternatively, MAFG may be upregulated in both THCA and COAD, resulting in CGI hypermethylation at a few MAFG binding sites, but some other factor may occur in COAD but not in THCA driving the extensive CGI hypermethylation in COAD.

Several mechanisms have been documented to support the consistent hypomethylation we observed in association with *TP53* mutations, across cancer types [48–51]. For example, in hepatocellular carcinoma, loss-of-function mutations in *TP53* allow pre-malignant cells to bypass senescence induced by global hypomethylation [48], which could explain the connection between *TP53* mutations and hypomethylation. In this study, we found that hypomethylated sites associated with *TP53* mutation are shared across cancer types and correspond to upregulated E2F-targets and genes involved in cell cycle regulation. This is interesting because the crosstalk between p53 and E2F pathways profoundly regulates the cell cycle [52]. Moreover, CpG methylation regulates E2F activity by preventing E2F family members from binding target promoters [53], supporting the correlation between *TP53*-associated hypomethylation at E2F targets and their upregulation. Upregulated E2F activity may promote cell proliferation, consistent with the association between *TP53* mutations and a high expression-based mitotic index in 9 cancer types found in this study (S3 Table). Therefore, the hypomethylation at E2F targets could regulate E2F activity or could simply represent the footprint of upregulated E2F activity due to *TP53* loss, yielding the association between *TP53* mutations and DNAm changes at E2F targets. In sum, several mechanisms, including hypomethylation-induced senescence and upregulated E2F activity, may underlie *TP53*-associated hypomethylation. Future research is needed to elucidate the role of hypomethylation in *TP53*-mutated tumors.

Whether the mutation-methylation associations found in this study are causal remains largely unresolved. However, the DNAm landscape can be affected by mutations in epigenetic modifying enzymes such as *SETD2*, the H3K36me3 writer [10]. In this study, many epigenetic modifying enzymes were implicated, including *PBRM1*, *BAP1*, *NSD1*, and *ARID1A*. In addition, mutations in driver genes may perturb the transcriptional circuitry. The perturbation can aberrantly activate or inactivate DNA binding proteins causing DNAm changes near their binding sites [54, 55]. This is evidenced by the recent finding that BRAF V600E and KRAS G13D mutations in COAD upregulate the transcription factors MAFG and ZNF304, respectively, resulting in targeted promoter CGI hypermethylation near MAFG and ZNF304 binding sites [13, 14]. The *TP53*-associated hypomethylation at E2F targets found in this study may also be explained in this way. Finally, several biological processes that can alter DNAm at specific sites have been documented recently—and driver gene mutations that promote these DNAm-altering processes may alter DNAm at affected sites. For example, cellular oxidative stress can produce hypermethylation at the promoters of low-expression genes [56], hypoxia can reduce TET activity, leading to hypermethylation at targeted sites [57], and cell proliferation can cause aberrant DNAm to accumulate in the promoters of polycomb group target CpGs [7].

Conversely, changes in DNAm can cause mutations in cancer. DNA hypomethylation, for example, leads to elevated mutation rates [58]. In addition, deamination of 5-methylcytosine can result in a C-to-T mutation. This could explain the occurrence of mutation hot spots at methylated CpG dinucleotides in *TP53* [59].

It is possible, of course, that no causal relationship exists between mutations and associated DNAm patterns. The associations observed may simply reflect the presence of specific DNAm patterns in the same tumor subtypes in which particular driver gene mutations are enriched or depleted. However, the absence of a causal relationship does not necessarily mean that mutations and methylation alterations occur in the same subtype merely by chance. In the previously mentioned example, DNA hypomethylation triggers *TP53*-mediated senescence, and hepatocellular carcinoma emerges when senescence is bypassed due to later *TP53* loss [48]. In this manner, positive selection for both the gene level and the DNAm level alterations could mechanistically link two non-causal events during tumorigenesis. This example suggests a mechanistic explanation that can be further investigated for mutation-methylation associations that lack clear causality.

Because mutation-methylation patterns may reflect important oncogenic characteristics, using these patterns to separate tumors into molecular subtypes could potentially aid treatment selection. In a proof of concept portion of this study, we successfully identified molecular subtypes in THCA and UCEC based on dominant driver gene–associated methylation patterns. These subtypes agreed with previous reports of subtypes defined by gene expression analyses [22, 26, 60]. In THCA, distinct DNAm patterns associated with *RAS*- and *BRAF*-mutated tumors corresponded to follicular and classical histological subtypes, respectively (Fig 4A) [60]. Likewise, the two UCEC subtypes characterized by *PTEN* and *TP53* mutations corresponded to the endometrioid-like and serous-like subtypes identified in TCGA analysis, respectively (Fig 4B) [22]. Moreover, TCGA classification indicated that the endometrioid-like subtype could be further subdivided into a microsatellite instability subtype (with a low frequency of *CTNNB1* mutations) and a low-copy-number subtype (with a high frequency of *CTNNB1* mutations). Consistent with this finding, in our study tumors with co-occurring *PTEN* and *CTNNB1* mutations displayed more hypomethylation (corresponding to the low-copy-number subtype) than tumors with *PTEN* mutations alone. Though we only attempted to identify subtypes in two cancer types, these results indicate that our mutation-methylation–based approach could be useful for identifying molecular subtypes in other cancer types as well.

The mutual exclusivity of the *NRAS*, *HRAS*, and *BRAF* mutations in THCA tumors has been interpreted to mean that these mutations must have interchangeable effects on MAPK signaling activation, the main cancer-driving event in papillary thyroid carcinomas [60]. Our analysis, however, highlights substantial differences in DNAm between *BRAF*-mutated vs. *NRAS*- and *HRAS*-mutated THCA tumors; moreover, the differences in DNAm appear to profoundly shape gene expression profiles, which may contribute to thyroid tumorigenesis. The JAK-STAT signaling pathway transmits information from extracellular signals to the nucleus, regulating genes involved in immunity and oncogenesis [61]. In this study, differential DNAm in six JAK and STAT family genes were found to correlate with their upregulation in *BRAF*-mutated tumors. Among them, *STAT3* has been studied in many cancer types, but its role in thyroid cancer is still debated. To date, one study has found a significant association between *STAT3* activation and metastatic disease in papillary thyroid carcinoma patients [62], whereas another has found that *STAT3* activation was inversely correlated with thyroid tumor growth [63]. In addition to JAK and STAT family genes, 9 metastatic genes were also differentially methylated and upregulated in *BRAF*-mutated tumors. Paired with the aggressiveness of *BRAF* vs. *RAS* mutation–positive thyroid tumors [64–66], our results support a connection between *BRAF* mutations, JAK-STAT signaling upregulation (including *STAT3* activation), and THCA metastasis, suggesting the role of *STAT3* and other JAK-STAT family genes in oncogenesis in THCA. There is also reason to be believe that the DNAm alterations in *NRAS*- and *HRAS*-mutated tumors have functional consequences for oncogenesis: Methylation changes in these tumors were linked to H_2_O_2_ overproduction, which can lead to DNA damage [46], activation of G-protein signaling via *GNAS* overexpression [43, 67], and activation of mTOR signaling via *PDK4* overexpression [60]. These differences in the molecular processes linked to different driver gene mutations may contribute to distinct pathways of tumorigenesis, yielding different prognoses and clinical phenotypes.

Our study has several limitations. First, all samples with mutations in the same MutSigCV-reported driver gene were classified together regardless of the mutation. For example, although the majority of *BRAF*-mutated samples carried the V600E mutation (25 out of 34 *BRAF*-mutated tumors carried BRAF V600E in COAD, 167/195 in SKCM, and 260/266 in THCA), this group also included a few non-V600E mutations. Different mutations in the same gene may be linked to different methylation patterns, therefore, introducing noise into our analysis and lowering our statistical power to detect mutation-methylation associations. Second, we examined individual associations between driver genes and methylation sites. However, combinatorial effects of driver gene mutations on methylation could exist, as several driver gene mutations typically co-occur in a given tumor. Third, we focused only on MutSigCV-reported driver genes and were limited to the information present in TCGA data. Although MutSigCV is one of the most reliable driver gene–detection tools available, limitations associated with the detection algorithm—paired with limitations imposed by the number of tumor samples available in TCGA—may have led us to miss methylation-altering mutations that occurred in unknown driver genes. Finally, tumor purity is a potential confounding factor in analyses of cancer data. Although we were not able to exclude the possibility of confounding by tumor purity, the mutation-methylation associations reported here were seen in cancer types for which most TCGA samples (>80%) were predicted to be of high purity (>70%), including GBM, KIRP, THCA, and UCEC [68]. Therefore, it seems unlikely that confounding by tumor purity level was extensive.

### Conclusions

This comprehensive pan-cancer analysis establishes a widespread connection between genomic and epigenomic alterations in cancer. The mutation-methylation relationships described here could potentially be used to identify tumor subtypes, thus aiding prognosis and treatment decisions. In addition, in the future, further analysis of methylation and expression data may identify driver gene mutation–induced methylation alterations that dysregulate genes/pathways that promote tumor growth. Such dysregulation could potentially be corrected by treating patients with agents that influence the DNA methylation landscape. Demethylating agents such as 5-aza-2'-deoxycytidine, for example, have been used to reactivate epigenetically silenced tumor suppressor genes and also to decrease overexpression of oncogenes [69, 70]. By contrast, the methyl donor S-adenosylmethionine has been shown to downregulate the oncogenes *c-MYC* and *HRAS*, inhibiting cancer cell growth [71]. In summary, in light of the connection between driver gene mutations and DNA methylation shown here, it will be important to further study how coordinated genomic and epigenomic alterations result in the hallmarks of cancer. A better understanding of the molecular mechanisms underlying cancer may help us identify factors that accelerate tumor onset, predict biomarkers for early diagnosis, and assess new therapeutic targets.

## Materials and methods

We downloaded data from all TCGA cancer types that had enough samples to support analyses (i.e. >100 samples for each data type: somatic mutation, DNA methylation 450K array, and RNA-Seq, according to the information posted on the UCSC cancer genome browser: https://genome-cancer.ucsc.edu/proj/site/hgHeatmap/) in 2015 and excluded cervical cancer and esophageal cancer whose molecular characterization had not been published by TCGA research network in 2016. We also excluded acute myeloid leukemia because we had reason to believe the mechanisms underlying this type of cancer could be very different from those underlying other solid tumors. This gave us the 18 cancer types: bladder urothelial carcinoma (BLCA), breast invasive carcinoma (BRCA), colon adenocarcinoma (COAD), glioblastoma multiforme (GBM), head and neck squamous cell carcinoma (HNSC), kidney renal clear cell carcinoma (KIRC), kidney renal papillary cell carcinoma (KIRP), liver hepatocellular carcinoma (LIHC), lung adenocarcinoma (LUAD), lung squamous cell carcinoma (LUSC), pancreatic adenocarcinoma (PAAD), prostate adenocarcinoma (PRAD), rectum adenocarcinoma (READ), skin cutaneous melanoma (SKCM), stomach adenocarcinoma (STAD), testicular germ cell tumors (TGCT), thyroid carcinoma (THCA), and uterine corpus endometrial carcinoma (UCEC). The number of tumor and normal samples (derived from tissue samples adjacent to tumors) for each cancer type is listed in Table 1.

### Data preprocessing

Exome-sequenced level 2 somatic mutation data were downloaded from TCGA’s data portal (https://tcga-data.nci.nih.gov/) on February 1, 2015.

TCGA level 3 DNA methylation array–based data (Illumina Infinium HumanMethylation450 BeadChip array) were downloaded from the UCSC Cancer Genomics Browser (https://genome-cancer.ucsc.edu) on October 26, 2015. DNA methylation levels were measured with β values. We normalized β values for type I and II probes using the β mixture quantile (BMIQ) method [72]. The following types of probes were removed from the analysis: (i) probes on the X and Y chromosomes, (ii) cross-reactive probes [73], (iii) probes near single nucleotide polymorphisms (SNPs), and (iv) probes with missing rates ≥90% across all samples for a given cancer type. A final set of 314,421 probes was analyzed for each cancer type.

Finally, TCGA level-3 gene expression data measured by log transformed (base 2) RSEM-normalized RNA-Seq (Illumina HiSeq) counts were downloaded from the UCSC Cancer Genomics Browser (https://genome-cancer.ucsc.edu) on November 4, 2015.

### Driver genes

We defined driver genes as those reported by MutSigCV2 [16] for each of the 18 cancer types; these data were downloaded from the Broad Institute of MIT & Harvard (http://firebrowse.org). Specifically, we analyzed genes that had reported q-values < 0.05 and that were mutated in at least five samples from each cancer type. The number of driver genes for the 18 cancer types is summarized in Table 1.

### Hyper- and hypomethylated probes

For each probe, we compared the distribution of methylation levels among tumor samples with that among normal samples using one-sided Wilcoxon rank-sum tests, one for each direction, stratified by cancer type. Each significant probe (q<0.05) was classified as either hypermethylated (methylation levels in tumor samples were greater than in normal samples) or hypomethylated (methylation levels in tumor samples were less than in normal samples).

### Principal component analysis

PCA was performed using the R package ‘pcaMethods,’ and missing values were imputed by probabilistic PCA. We wanted to analyze as many PCs as we could, to capture the majority of DNAm variation. However, the more PCs we analyze, the longer the computation time needed. We found that 5 PCs provided an ideal balance: the PCs captured the majority of mutation-methylation associations within a reasonable computation time. The top five PCs were computed within each cancer type for all probes and subsets of probes, including probes in CGIs, the SSs around CGIs (the 4-kb regions flanking CGIs), and open sea regions (CpGs outside CGIs and SSs). The probe sets (CGIs, SSs, and open sea regions) were further stratified by hyper- and hypomethylation status. CGIs, SSs, and open sea regions were defined in the Illumina 450K array annotation file.

### HyperZ and HypoZ indices

HyperZ and HypoZ indices were computed for each tumor sample within a cancer type. The HyperZ and HypoZ indices were introduced by Yang et al. [33] to measure the level of overall CGI hypermethylation and open sea hypomethylation, respectively.

### Association between driver gene mutations and DNA methylation

We analyzed somatic mutations at the gene level. A driver gene was classified as either mutated (any mutations) or not mutated (no mutations) for each tumor sample. Associations between driver gene mutations and methylation were tested using the two-sided Wilcoxon rank-sum test. To evaluate driver gene–PC associations, the test was performed for every driver gene and each of the top five methylation PCs; samples were ranked based on their coordinates on the PC, and the mutated cohort was compared with the non-mutated cohort. To evaluate site-specific associations, the test was performed for every possible driver gene–probe pair. Here, samples were ranked based on their β values at the probe, and the mutated cohort was compared with the non-mutated cohort. Finally, we performed the same association test for every driver gene and the HyperZ and HypoZ indices, to identify driver genes potentially associated with genome-wide CGI hypermethylation and open sea hypomethylation. In each case, q-values were computed by correcting for all tests performed for a given cancer type [74]. Associations were considered significant at q<0.05. For associations between every gene-probe pair, the empirical false discovery rate was also estimated by permuting the mutation status for every driver gene. The results showed that the empirical false discovery rate was controlled (<0.05) at the theoretical cutoff (q<0.05) for each cancer type (S8 Fig).

### Differential expression analysis of thyroid carcinoma molecular subtypes

First, we visually identified two THCA molecular subtypes based on driver gene mutations and DNA methylation patterns (Fig 4A): (i) *BRAF*-mutated tumors (the *BRAF* group) and (ii) *NRAS*- and *HRAS*-mutated tumors (the *NRAS-HRAS* group). Next, we assembled four differentially expressed gene sets (up- or downregulated in one group but identical or regulated in the opposite direction in another group, compared with normal samples). The search was restricted to genes whose aberrant expression levels coincided with hyper- or hypomethylated probes associated with *BRAF*, *HRAS*, and *NRAS* mutations (see section below). Using a hypergeometric model, genes in each of the four sets were tested for enrichment by 1,330 canonical pathways collected by MsigDB [75]. The highly transcribed, differential genes were defined by expression levels that were greater than 10 log_2_ RSEM and at least double those of normal samples.

### Association between driver gene–associated aberrant methylation and aberrant gene expression in thyroid carcinomas

We sought genes whose aberrant expression was correlated with aberrant methylation in the presence of *BRAF*, *HRAS*, or *NRAS* mutations. First, we identified probes that fell within 1,500 bp of the transcription start site or within gene bodies, and whose β values were significantly correlated with expression levels of the corresponding gene. Because a methylated CpG may up- or downregulate gene expression in a context-dependent manner [70, 76, 77], we computed the significance of the Spearman correlation between each individual probe’s methylation and the expression level of its corresponding gene. We considered gene-probe pairs significant at q<0.05. Second, we analyzed the association between β values and *BRAF*, *HRAS*, and *NRAS* mutation status for all probes using the two-sided Wilcoxon rank-sum test. For each driver gene, aberrantly methylated probes (q<0.05) were classified as hyper- or hypomethylated relative to normal samples. Third, we integrated the results from the first and second steps to identify aberrantly methylated probes whose methylation levels were significantly correlated with the expression levels of their corresponding genes for each group of *BRAF*-, *HRAS*-, and *NRAS*- mutated samples. Finally, for each group of mutated samples, significantly up- or downregulated genes were identified from the aberrant methylation-matched genes identified in the third step, using the two-sided Wilcoxon rank-sum test relative to the expression levels of normal samples (q<0.05). For example, when we looked for genes that were upregulated in *BRAF*-mutated samples but exhibited no change or were downregulated in *HRAS*- and *NRAS*-mutated samples, we restricted the search to genes that were hyper- (or hypo-) methylated in *BRAF*-mutated samples but exhibited no change or were hypo- (or hyper-) methylated in *HRAS*- and *NRAS*-mutated samples in the third step. Thus, for each driver gene, we obtained a list of aberrantly methylated probes associated with genes that were aberrantly expressed.

## Supporting information

S1 Text Driver gene mutations, methylation patterns, and cell proliferation are associated. (DOCX)

S1 Fig Driver gene–methylation associations across 18 cancer types (*rows*), stratified by CpG subsets (*columns*). The numbers (1–5) indicate the top five principal components (PCs) for each probe set, whereas the colors show the significance of the strongest association between each methylation PC and any driver gene. The probe sets represent methylome (all probes), CpG island (CGI) probes, shore and shelf (SS) probes, and open sea probes, further stratified by hyper- and hypomethylation status. For glioblastoma (GBM), stomach adenocarcinoma (STAD), skin cutaneous melanoma (SKCM), and testicular germ cell tumor (TGCT), there were not enough normal samples to compute associations for hyper-/hypomethylated probes (shown in dark grey).
(TIFF)

S2 Fig DNAm variation is correlated with mutated driver genes and mitotic indices. In 15 of 18 cancer types examined, mutated driver genes were associated with one or more of the top five methylation PCs, shown as rows. The three driver genes most significantly associated with each PC are reported. Driver genes associated with the negative extreme of the PC are in blue, whereas associations with the positive extreme are in red. Background colors indicate correlation status (q<0.05; Spearman correlation) with two mitotic indices. Light green indicates a significant correlation with the DNAm-based index (epiTOC [7]); light yellow a significant correlation with the expression-based index; orange indicates correlations with both indices, in the same direction; and brown indicates correlations with both indices, in opposite directions. The methylation PC–driver gene association shown here is identical to that in Fig 1. See Table 1 for cancer type abbreviations.
(TIFF)

S3 Fig Driver gene mutations are significantly associated with DNA methylation variation in various cancers, after removing probes correlated with the DNAm-based mitotic index. The DNAm-based mitotic index, called epiTOC (for epigenetic Timer Of Cancer), was used to approximate the cell proliferation rate in cancer [7]. In 11 of 18 cancer types examined, driver gene mutations were associated with one or more of the top five epiTOC-uncorrelated methylation principal components (PCs). Shown is a grid depicting the three driver genes most significantly associated with each PC. A gene name in blue indicates that mutations in that gene were significantly associated with the negative extreme of the PC, whereas red indicates a gene was associated with the positive extreme of the PC. For each PC, a white background indicates no correlation with epiTOC was present (q<0.05; Spearman correlation). See Table 1 for cancer type abbreviations.
(TIFF)

S4 Fig Distribution of driver gene–associated methylation probes throughout the genome in kidney renal clear cell carcinoma (KIRC). (A) Chromosomes 1 to 22 are plotted on a circle, with each chromosome plotted proportional to chromosome length and labeled in the outermost track. The 14 inner tracks correspond to all 14 driver genes in KIRC; gene names and the number of associated probes for each are shown. For each driver gene, associated probes are plotted as line segments in the corresponding track at the appropriate chromosome location. The chromosome length scale is labeled for chromosome 1 (a major interval indicates 90 Mb). (B) A heat map shows driver gene mutation profiles across KIRC tumor samples.
(TIFF)

S5 Fig Genomic distribution of the probes that are positively and negatively associated with *RNF43*. The bar plot shows the percentage of probes falling in 11 different annotated genomic regions for *RNF43*, for all probes analyzed (labeled array) and after stratifying by the direction of association (positive or negative). The genomic distribution of probes was obtained with ChIPseeker [78].
(TIFF)

S6 Fig Probes that are negatively associated with *TP53* are shared across cancer types. A bar plot shows the number of probes negatively associated with *TP53* (y-axis in log10 scale; number of probes is also indicated at the top of each bar) in at least 1 to 7 cancer types (x-axis).
(TIFF)

S7 Fig Methylation subtypes identified by using the top 1% most variable probes in thyroid carcinoma (THCA) and uterine corpus endometrial carcinoma (UCEC). Heat maps for (A) THCA and (B) UCEC depict hierarchical clustering of methylation values of the top 1% most variable probes (based on variance across tumor samples; 3,145 probes in total). Each column represents a sample, and each row represents a probe. Mutation status is shown in the upper sidebar. Sidebars on the left indicate CpG subset and average methylation levels across normal samples. The subtypes identified and their corresponding mutation status are similar to those shown in Fig 4.
(TIFF)

S8 Fig Empirical false discovery rates (FDRs) are controlled by theoretical cutoffs at q = 0.05 for site-specific associations. Site-specific associations were tested between every driver gene and every probe. Significant associations were called at a theoretical FDR (q-value) < 0.05 for each cancer type. The empirical FDR (y-axis) was estimated for the theoretical cutoff (q = 0.05) by permuting mutation status for each driver gene in each cancer type (*column*). Here, all points are below the line (empirical FDR = 0.05), indicating that empirical FDRs are controlled by the theoretical cutoffs.
(TIFF)

S1 Table Driver genes associated with any of the top five principal components across 18 cancer types (q<0.05; Wilcoxon rank-sum test). (XLSX)

S2 Table Counts for driver gene–methylation probe associations for 18 cancer types (q<0.05; Wilcoxon rank-sum test), stratified by CpG subset [methylome (all probes), CpG island probes, shores and shelves probes, and open sea probes] as well as hypo- or hypermethylated status (+: positive associations, -: negative associations); epigenetic modifying enzymes are annotated. (XLSX)

S3 Table Associations between mutated driver genes and DNAm- and expression-based mitotic indices. (DOCX)

S4 Table Number of *TP53*-associated probes in 18 cancer types (q<0.05; corrected for associations between all probes and *TP53* within each cancer type; Wilcoxon rank-sum test). (XLSX)

S5 Table Canonical pathways and regulatory gene sets enriched with up-/downregulated genes shared across at least two cancer types that correspond to DNAm changes in *TP53*-mutated tumors. (XLSX)

S6 Table Sorted median expression levels of genes differentially expressed (relative to normal samples) in the two thyroid carcinoma molecular subtypes identified in Fig 4A: *BRAF*-mutated vs *NRAS*-/*HRAS*-mutated; each Excel tab separates genes by the direction of expression dysregulation (*up* or *down*) and the group of mutated samples showing the expression dysregulation (*BRAF* or *HRAS*/*NRAS*). (XLSX)

S7 Table Pathways enriched with genes dysregulated in the two thyroid carcinoma molecular subtypes identified in Fig 4A: *BRAF*-mutated vs *HRAS*/*NRAS*-mutated, stratified by the direction of expression dysregulation (*up* or *down*) and the mutant group (*BRAF* or *HRAS*/*NRAS*). (XLSX)
