# Supplementary material for: Significant associations between driver gene mutations and DNA methylation alterations across many cancer types
Source: PLoS Comput Biol. 2017 Nov 10;13(11):e1005840. doi: 10.1371/journal.pcbi.1005840 (PMC5709060; doi:10.1371/journal.pcbi.1005840)
Supplement: S1 Text — (DOCX) [file pcbi.1005840.s001.docx]

**S1 Text**

**Driver gene mutations, methylation patterns, and cell proliferation are associated**

Cell proliferation was proposed to be an important factor driving DNAm changes in cancer [1]. This was demonstrated with a DNAm-based mitotic index where the average methylation level across 385 CpGs in the Illumina 450K array was used to approximate the cell proliferation rate in cancer, called epiTOC (epigenetic Timer Of Cancer). In many cancer types, epiTOC is positively correlated with an expression-based mitotic index (the average mRNA expression level across 9 mitotic genes: *CDKN3*, *ILF2*, *KDELR2*, *RFC4*, *TOP2A*, *MCM3*, *KPNA2*, *CKS2*, and *CDC2*) [1] (Pearson correlation for BLCA=0.22; BRCA=0.33; COAD=0.39; GBM=0.39; HNSC=0.22; KIRC=0.45; KIRP=0.56; LIHC=0.2; LUAD=0.17; ^*^LUSC=0.1; PAAD=0.47; PRAD=0.49; READ=0.24; ^*^SKCM=0.05; STAD=0.31; ^*^TGCT=0.07; THCA=0.34; UCEC=0.28; ^*^p>0.05). We calculated both mitotic indices for all tumor types. We found these two indices were correlated with many of the top 5 PCs across cancer types (q<0.05; Spearman correlation) (S2 Fig). In general, epiTOC correlated with more PCs (n=65) than the expression-based index (n=33), suggesting that epiTOC likely explains DNAm variation in a greater extent. Across 18 cancer types, 25 PCs were correlated with both indices with 6/25 PCs correlated in opposite direction (S2 Fig). To study whether the DNAm variation explained by mutated driver genes can be also explained by epiTOC, we removed epiTOC-correlated methylation probes (p<0.05; Pearson correlation) and repeated the analysis. We found that many methylation PC associated-driver genes remained in many cancer types (S3 Fig), indicating that the mutation-methylation association cannot be totally explained by epiTOC scores. To elucidate the relationship between the two indices and mutated driver genes, we associated the presence of mutations in each driver gene with each index (q<0.05; Wilcoxon rank sum test). We found that the two indices were inconsistent in association with mutated driver genes in most cancer types (S3 Table). For example, *TP53*-mutated tumors were positively associated with the expression-based mitotic index in 9 cancer types and none displayed negative association in any type. In contrast, *TP53*-mutated tumors were negatively associated with epiTOC in HNSC and UCEC but none displayed positive association. Thus epiTOC and the expression-based mitotic index are inconsistent and could be measuring two different properties of these tumor cells. Despite their inconsistency, using the cell proliferation rate approximated by either index, our results indicate that methylome patterns in cancer are correlated with the presence of mutated driver genes and cell proliferation but the mutation-methylation association found in this study cannot be explained by cell proliferation alone.

**Reference**

1. Yang Z, Wong A, Kuh D, Paul DS, Rakyan VK, Leslie RD, et al. Correlation of an epigenetic mitotic clock with cancer risk. Genome Biol. 2016; 17(1):205. https://doi.org/10.1186/s13059-016-1064-3 PMID: 27716309
