# Supplementary figures and images for: Significant associations between driver gene mutations and DNA methylation alterations across many cancer types
Source: PLoS Comput Biol. 2017 Nov 10;13(11):e1005840. doi: 10.1371/journal.pcbi.1005840 (PMC5709060; doi:10.1371/journal.pcbi.1005840)

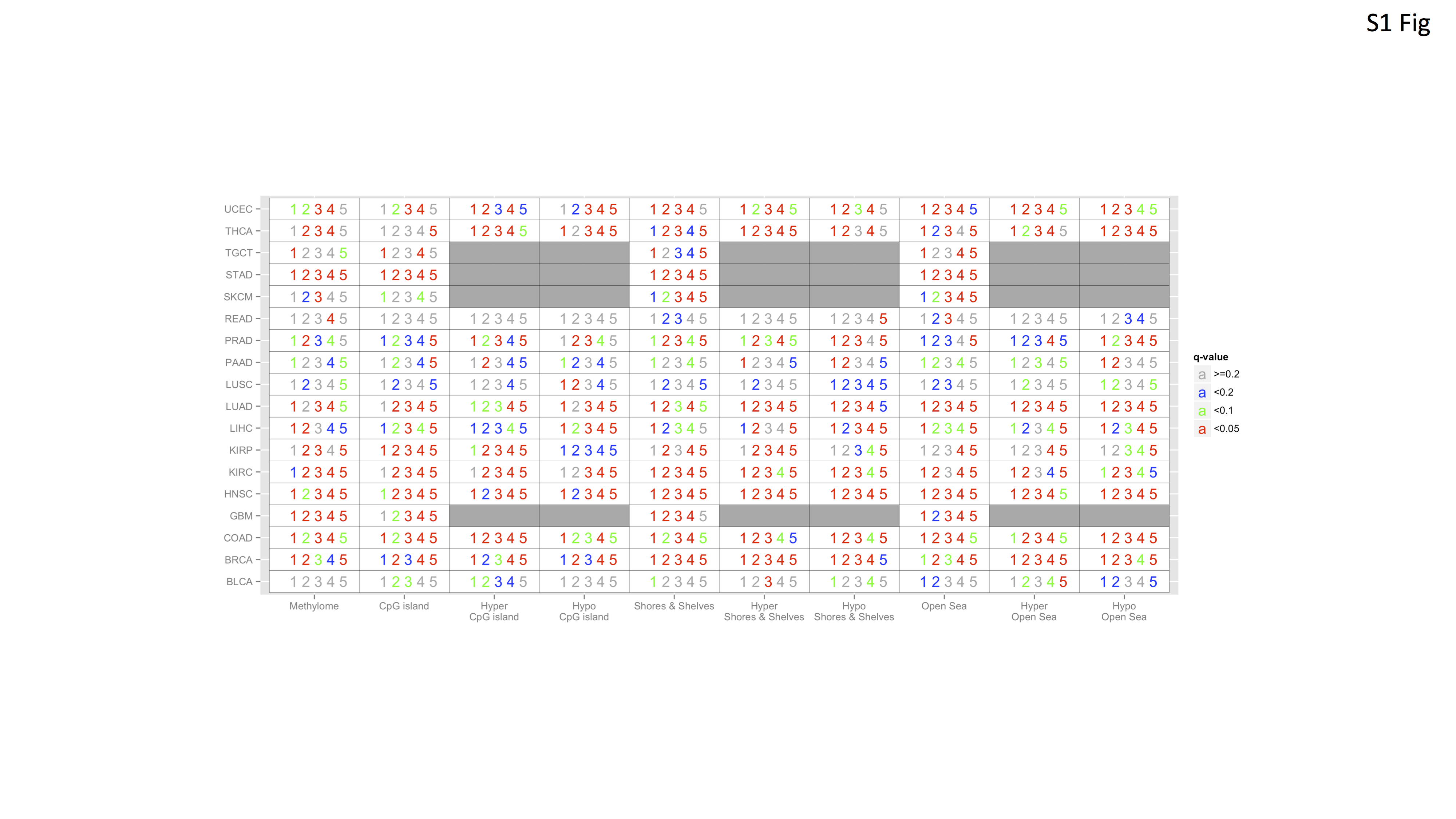

Supplement: S1 Fig — The numbers (1–5) indicate the top five principal components (PCs) for each probe set, whereas the colors show the significance of the strongest association between each methylation PC and any driver gene. The probe sets represent methylome (all probes), CpG island (CGI) probes, shore and shelf (SS) probes, and open sea probes, further stratified by hyper- and hypomethylation status. For glioblastoma (GBM), stomach adenocarcinoma (STAD), skin cutaneous melanoma (SKCM), and testicular germ cell tumor (TGCT), there were not enough normal samples to compute associations for hyper-/hypomethylated probes (shown in dark grey). (TIFF) [file pcbi.1005840.s002.tiff]

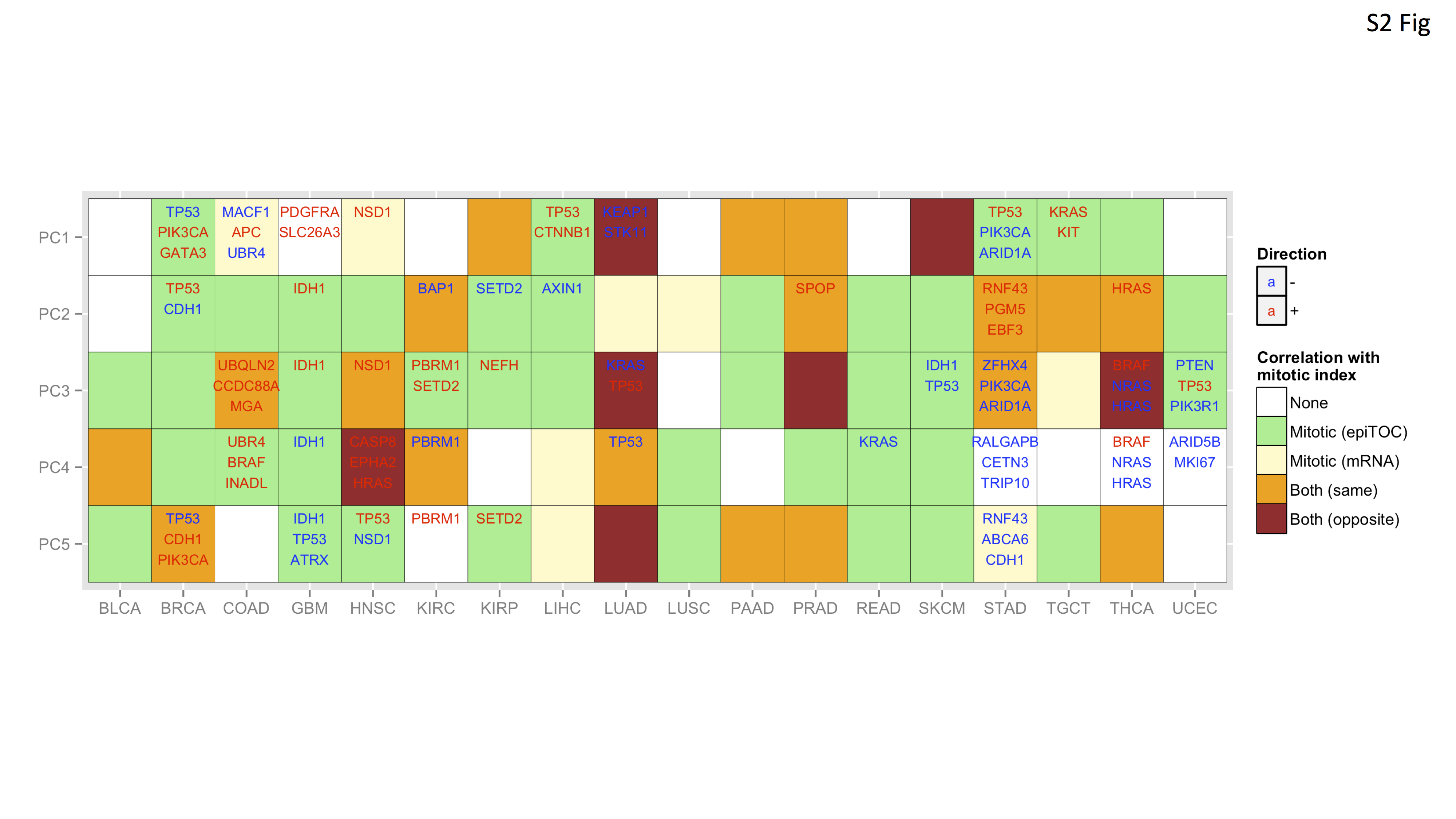

Supplement: S2 Fig — In 15 of 18 cancer types examined, mutated driver genes were associated with one or more of the top five methylation PCs, shown as rows. The three driver genes most significantly associated with each PC are reported. Driver genes associated with the negative extreme of the PC are in blue, whereas associations with the positive extreme are in red. Background colors indicate correlation status (q<0.05; Spearman correlation) with two mitotic indices. Light green indicates a significant correlation with the DNAm-based index (epiTOC [7]); light yellow a significant correlation with the expression-based index; orange indicates correlations with both indices, in the same direction; and brown indicates correlations with both indices, in opposite directions. The methylation PC–driver gene association shown here is identical to that in Fig 1. See Table 1 for cancer type abbreviations. (TIFF) [file pcbi.1005840.s003.tiff]

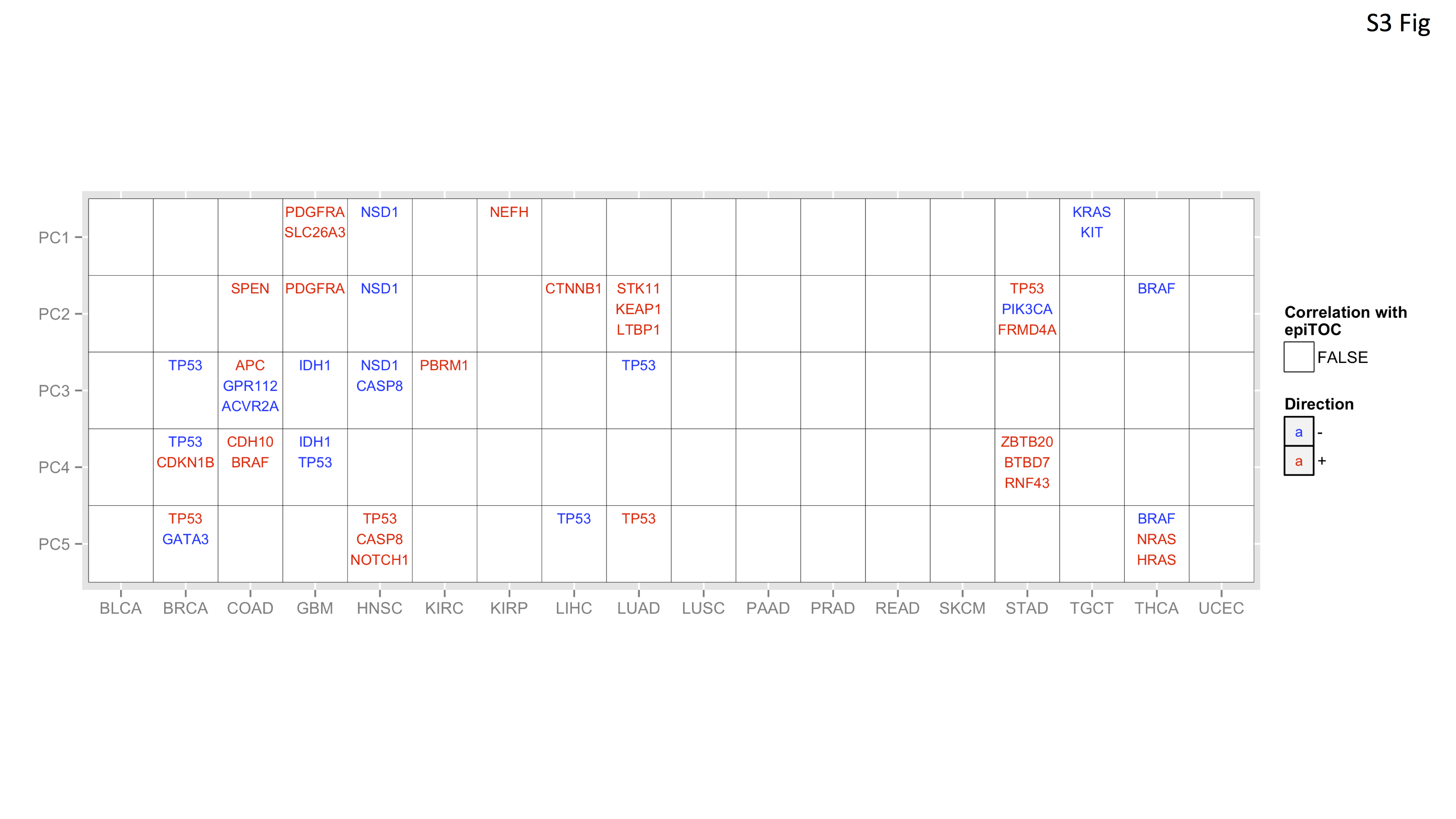

Supplement: S3 Fig — The DNAm-based mitotic index, called epiTOC (for epigenetic Timer Of Cancer), was used to approximate the cell proliferation rate in cancer [7]. In 11 of 18 cancer types examined, driver gene mutations were associated with one or more of the top five epiTOC-uncorrelated methylation principal components (PCs). Shown is a grid depicting the three driver genes most significantly associated with each PC. A gene name in blue indicates that mutations in that gene were significantly associated with the negative extreme of the PC, whereas red indicates a gene was associated with the positive extreme of the PC. For each PC, a white background indicates no correlation with epiTOC was present (q<0.05; Spearman correlation). See Table 1 for cancer type abbreviations. (TIFF) [file pcbi.1005840.s004.tiff]

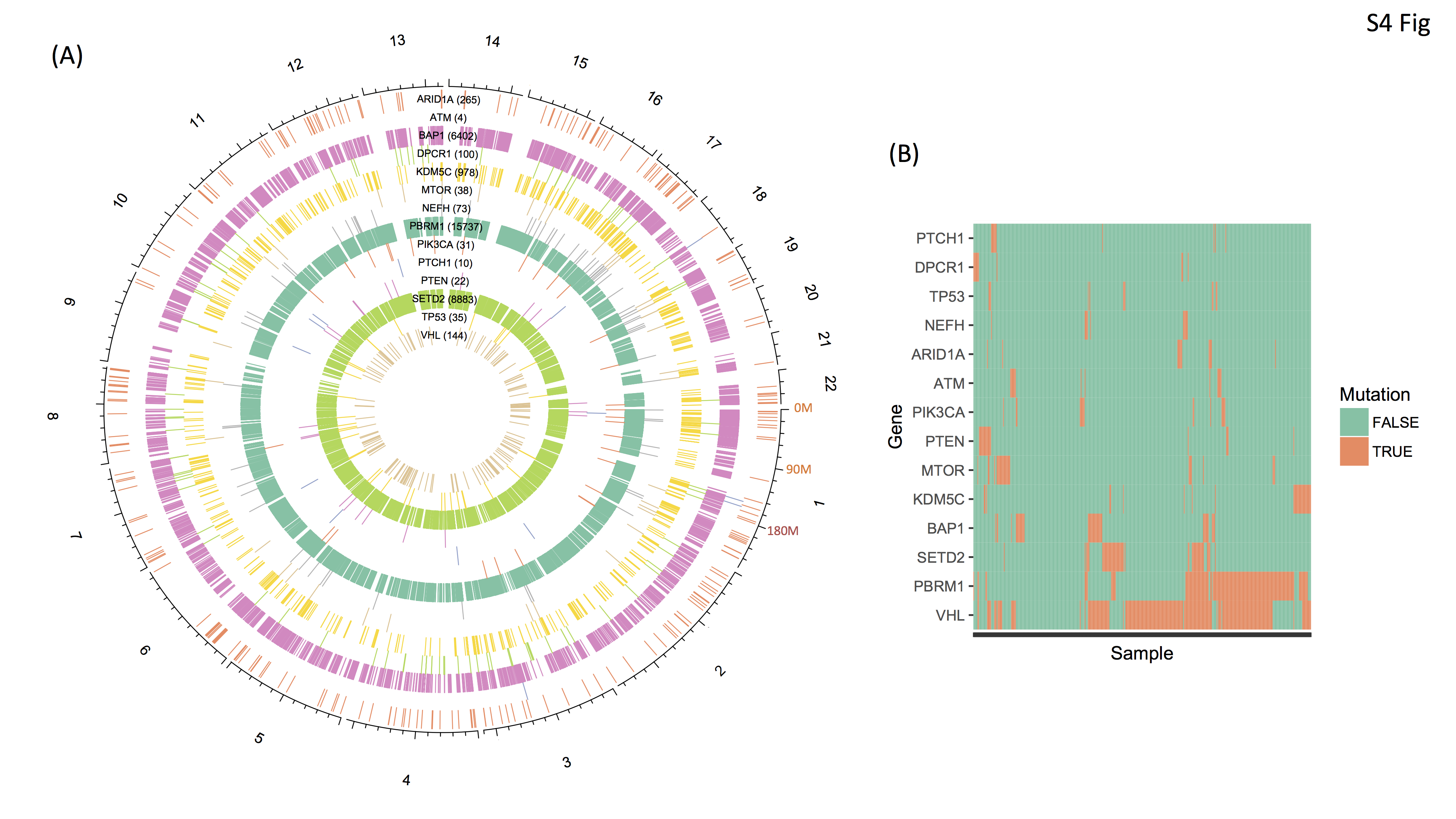

Supplement: S4 Fig — (A) Chromosomes 1 to 22 are plotted on a circle, with each chromosome plotted proportional to chromosome length and labeled in the outermost track. The 14 inner tracks correspond to all 14 driver genes in KIRC; gene names and the number of associated probes for each are shown. For each driver gene, associated probes are plotted as line segments in the corresponding track at the appropriate chromosome location. The chromosome length scale is labeled for chromosome 1 (a major interval indicates 90 Mb). (B) A heat map shows driver gene mutation profiles across KIRC tumor samples. (TIFF) [file pcbi.1005840.s005.tiff]

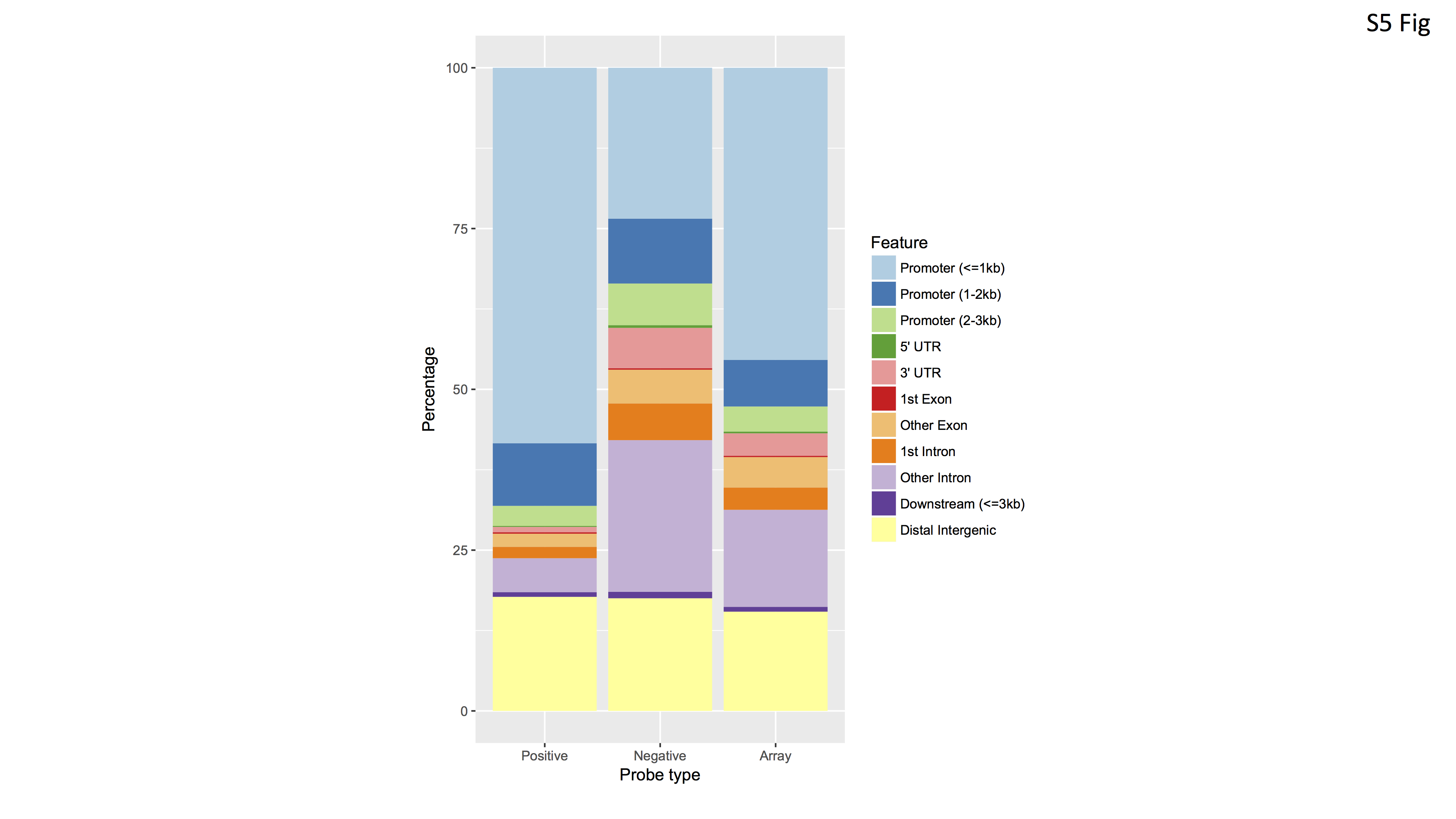

Supplement: S5 Fig — The bar plot shows the percentage of probes falling in 11 different annotated genomic regions for RNF43, for all probes analyzed (labeled array) and after stratifying by the direction of association (positive or negative). The genomic distribution of probes was obtained with ChIPseeker [78]. (TIFF) [file pcbi.1005840.s006.tiff]

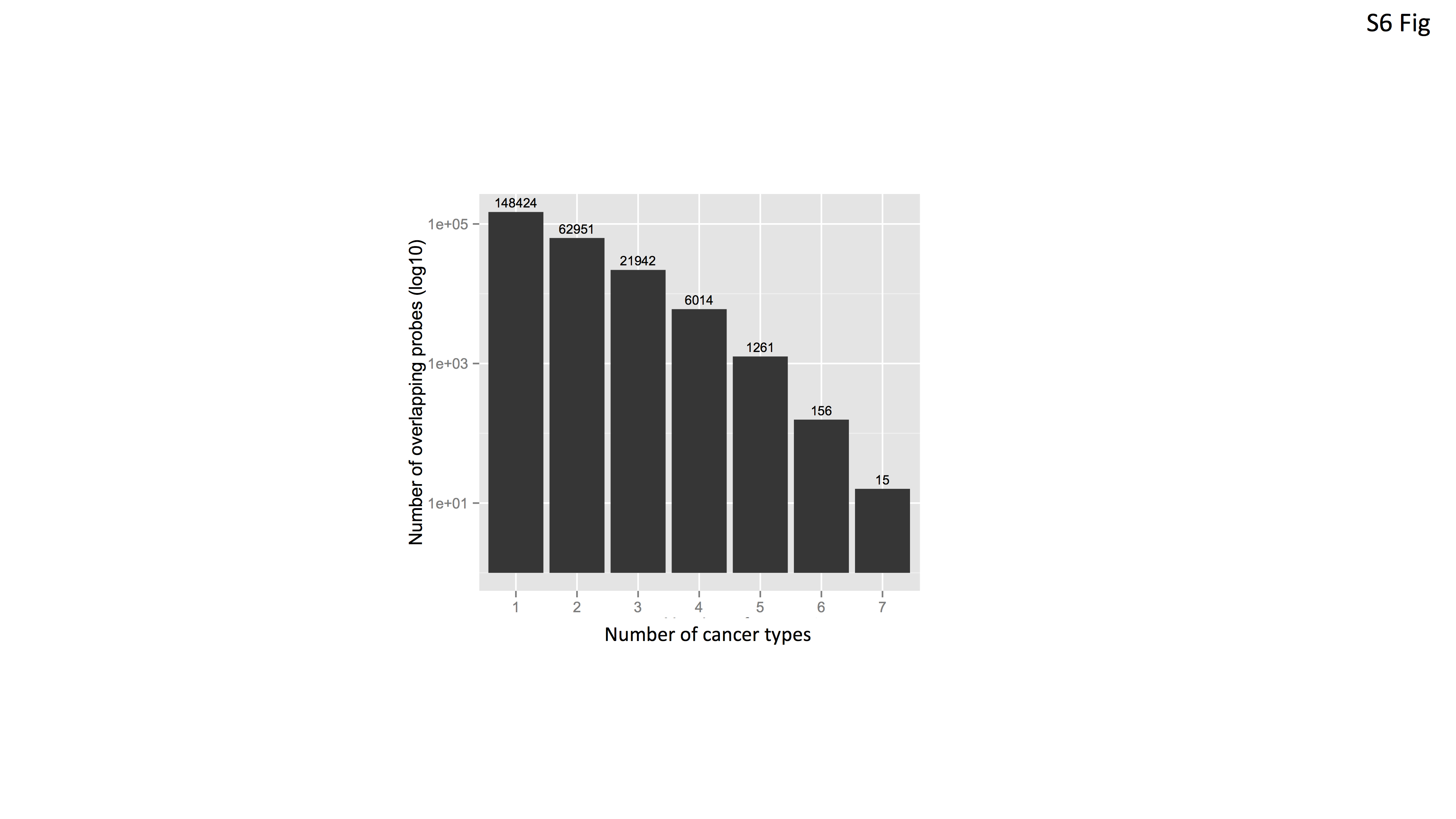

Supplement: S6 Fig — A bar plot shows the number of probes negatively associated with TP53 (y-axis in log10 scale; number of probes is also indicated at the top of each bar) in at least 1 to 7 cancer types (x-axis). (TIFF) [file pcbi.1005840.s007.tiff]

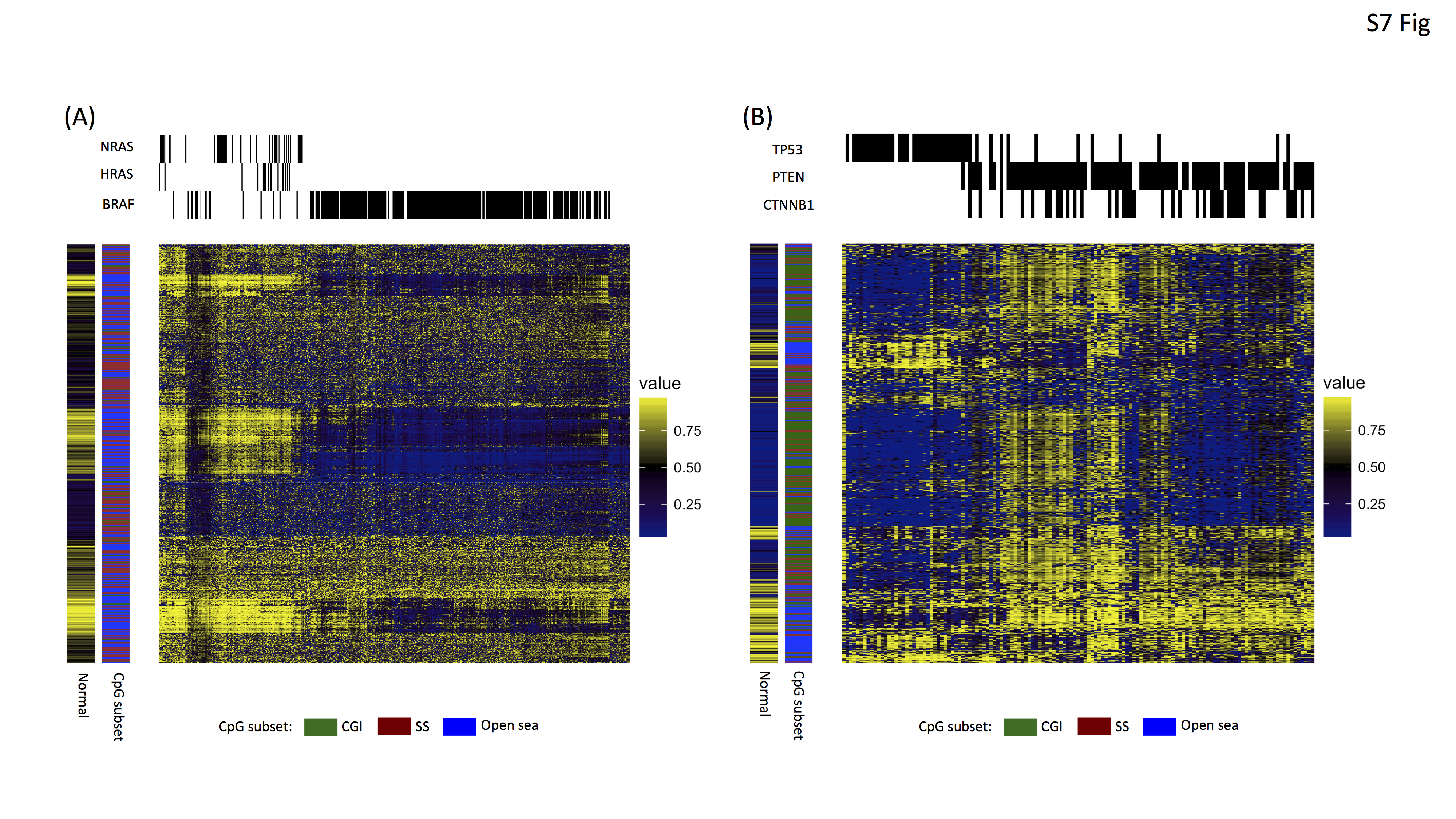

Supplement: S7 Fig — Heat maps for (A) THCA and (B) UCEC depict hierarchical clustering of methylation values of the top 1% most variable probes (based on variance across tumor samples; 3,145 probes in total). Each column represents a sample, and each row represents a probe. Mutation status is shown in the upper sidebar. Sidebars on the left indicate CpG subset and average methylation levels across normal samples. The subtypes identified and their corresponding mutation status are similar to those shown in Fig 4. (TIFF) [file pcbi.1005840.s008.tiff]

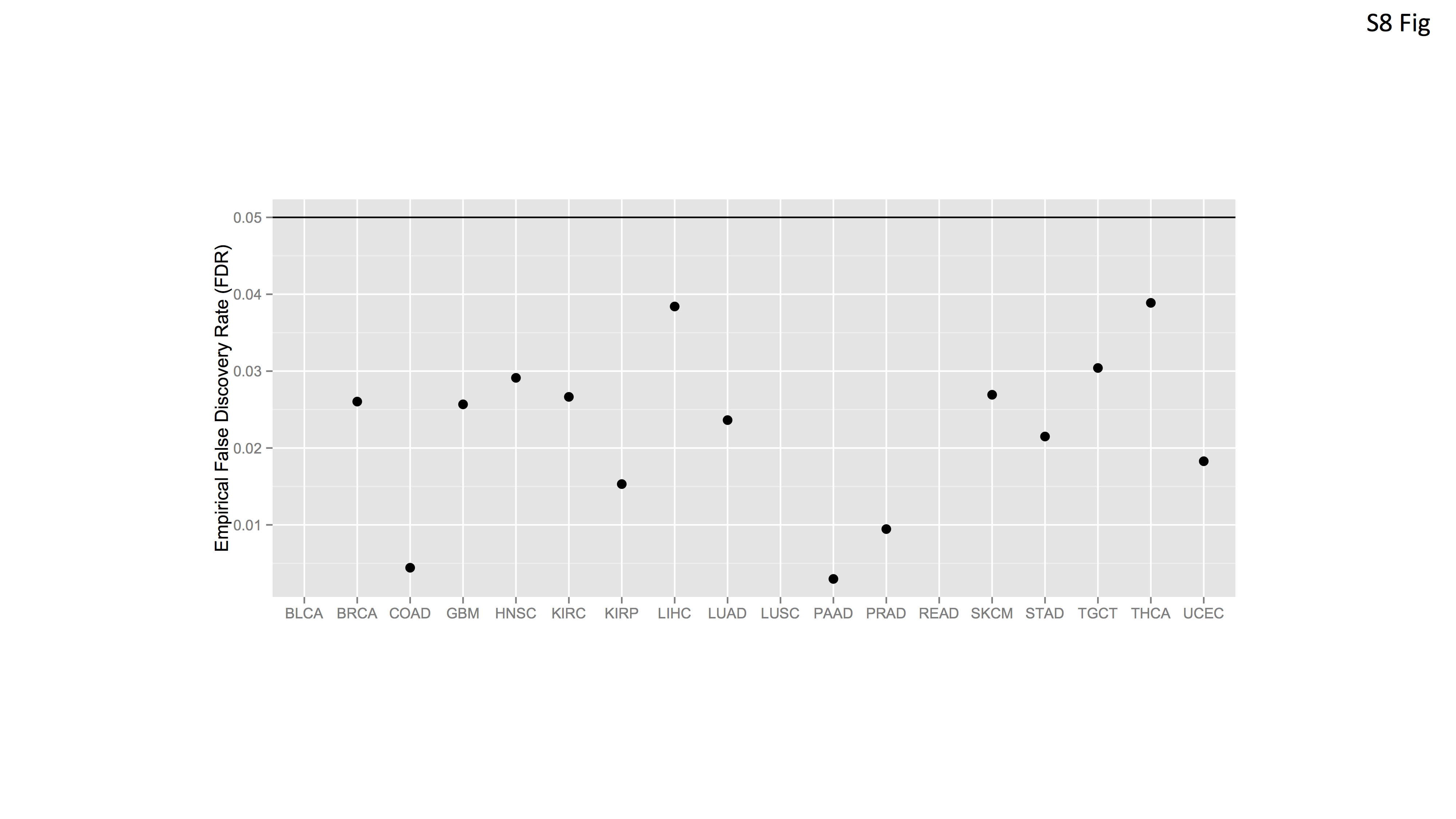

Supplement: S8 Fig — Site-specific associations were tested between every driver gene and every probe. Significant associations were called at a theoretical FDR (q-value) < 0.05 for each cancer type. The empirical FDR (y-axis) was estimated for the theoretical cutoff (q = 0.05) by permuting mutation status for each driver gene in each cancer type (column). Here, all points are below the line (empirical FDR = 0.05), indicating that empirical FDRs are controlled by the theoretical cutoffs. (TIFF) [file pcbi.1005840.s009.tiff]
