# Supplementary material for: Significant associations between driver gene mutations and DNA methylation alterations across many cancer types
Source: PLoS Comput Biol. 2017 Nov 10;13(11):e1005840. doi: 10.1371/journal.pcbi.1005840 (PMC5709060; doi:10.1371/journal.pcbi.1005840)
Supplement: S3 Table — (DOCX) [file pcbi.1005840.s012.docx]

**S3 Table. Associations between mutated driver genes and DNAm- and expression-based mitotic indices^abd^**

| **Cancer type** | **High proliferation (DNAm)** | **High proliferation (expression)** | **Low proliferation (DNAm)** | **Low proliferation (expression)** |
| --- | --- | --- | --- | --- |
| BLCA |  | RB1, **TP53** |  |  |
| BRCA | **CDH1**, FOXA1 | BCORL1, RB1, **TP53** |  | **CDH1**, MAP3K1, PIK3CA |
| HNSC | CASP8, CTCF, EPHA2, HRAS |  | **TP53** |  |
| KIRC | BAP1, **PBRM1**, SETD2 | BAP1, PTEN, SETD2, **TP53** |  | **PBRM1**, VHL |
| KIRP | NF2, SETD2 | SETD2 |  |  |
| LIHC |  | RB1, **TP53** |  |  |
| LUAD | **KRAS** | COL5A2, GLDC, **KEAP1**, MYH7, RB1, SMARCA4, STRA8, **TP53** | **KEAP1** | **KRAS** |
| LUSC |  | **TP53** |  | SLC28A1 |
| PAAD | CDKN2A, KRAS |  |  |  |
| PRAD | SPOP | **TP53** |  |  |
| SKCM |  | ALPK2, IL5RA, NBPF1, PTEN, **TP53**, XIRP2 |  |  |
| STAD | 189 driver genes (not including TP53) | 52 driver genes (including **TP53**) |  | CDH1, RHOA |
| TGCT | MLLT3, MUC6 |  |  | KIT |
| THCA |  | BRAF |  | NRAS |
| UCEC | ARID1A, ARID5B, BCOR, CUX1, KRAS, PIK3R1, PTEN, SIN3A, ZFHX3 | NA^c^ | **TP53** | NA^c^ |

^a^ Association is computed by Wilcoxon rank sum test at q<0.05 for each cancer.

^b^ TP53 and inconsistent genes (associated with both indices in opposite directions) are highlighted in bold

^c^ Too few (n=5) with both gene expression and mutation data for UCEC.

^d^ No association shown for COAD, GBM, and READ
